# Evaluating safety and quality of robotic-assisted gastric cancer surgery: meta-analysis and meta-regression

**DOI:** 10.1093/bjsopen/zraf126

**Published:** 2025-11-24

**Authors:** Riadh Salem, Wing K Chou, Lorenzo Giorgi, Sivesh K Kamarajah, Sheraz R Markar

**Affiliations:** Nuffield Department of Surgical Sciences, University of Oxford, Oxford, UK; Nuffield Department of Surgical Sciences, University of Oxford, Oxford, UK; Nuffield Department of Surgical Sciences, University of Oxford, Oxford, UK; Department of Applied Health Sciences, University of Birmingham, Birmingham, UK; Nuffield Department of Surgical Sciences, University of Oxford, Oxford, UK; Department of Surgery, Churchill Hospital, Oxford University Hospitals NHS Trust, Oxford, UK

**Keywords:** robotic surgical procedures, gastrectomy, stomach neoplasms, quality assurance, patient safety

## Abstract

**Background:**

Robotic-assisted surgery is expanding globally. The UK’s National Institute for Health and Care Excellence recently cautioned due to a paucity of high-quality evidence. To address this, a systematic review, meta-analysis, and meta-regression were undertaken to evaluate the quality and safety of robotic-assisted gastrectomy (RAG) *versus* conventional approaches for gastric cancer.

**Methods:**

Systematic searches were conducted on MEDLINE, Embase, Web of Science, and Scopus (2 May 2025) for studies comparing RAG to open or laparoscopic gastrectomy up to 30 April 2025. Primary outcomes were Clavien–Dindo grade ≥ II complications (CD ≥ II; safety) and margin-positive resections (quality). Risk of bias was assessed using the Risk Of Bias In Non-randomized Studies of Interventions and Cochrane Risk of Bias v2.0 tools. Heterogeneity and evidence certainty were evaluated using meta-regression and GRADE assessment.

**Results:**

In all, 90 studies (65 296 patients) were included; only three studies were randomized clinical trials and 72 were from East Asia. In 44 studies (12 102 patients) RAG was associated with significantly lower CD ≥ II complications (odds ratio (OR) 0.74; 95% confidence interval (c.i.) 0.64 to 0.86); heterogeneity was low (*I*^2^ = 21.4%). Seven studies had a low risk of bias. From 35 studies on margin status, RAG had fewer R1 resections (OR 0.74; 95% c.i. 0.51 to 1.07); heterogeneity was moderate (*I*^2^ = 34.0%). Adoption year, industry funding, extent of resection, and tumour stage were identified as sources of heterogeneity. Three studies were at low risk of bias. Certainty was very low for both outcomes.

**Conclusion:**

Although there may be potential benefits of robotic-assisted surgery, cautious adoption is warranted given the current uncertainty. Safe adoption requires standardized training, competency benchmarks, and limiting industry involvement. High-quality evaluation through randomized trials and parallel health economics is urgently needed to inform future policy and practice.

## Teaser

This systematic review and meta-analysis of 90 studies (65 296 patients) found very low-certainty evidence suggesting possible benefits of robotic-assisted gastrectomy over conventional surgery. The overall quality of evidence was poor, with most studies non-randomized and at moderate risk of bias. Meta-regression identified industry involvement as a significant source of heterogeneity, highlighting the need for independent evaluation and cautious adoption.

## Introduction

Globally, there has been rapid expansion of robotic platforms, driven largely by the surgical robotics industry^1^. Recent evidence suggests an almost eight-fold increase in the use of robotic platforms between 2012 and 2018^2^. Despite this growing adoption in large tertiary centres, health systems around the world struggle to adopt such practices owing to debates on the potential benefits and return on substantial investment^3–5^. Recently, the National Institute for Heath and Care Excellence (NICE) based in the UK undertook a health technology assessment of robotic surgery in soft tissue procedures, including colorectal, upper gastrointestinal, and urological cancer surgeries^6^. However, uncertainty remains around these areas, which require high-quality evidence to justify the widespread use of robotic surgery in the National Health Service (NHS). This evidence includes robust clinical and health economic evaluation, as well as surgical quality assurance with learning curve assessment^7^.

Among soft tissue procedures, surgery for gastric cancers is particularly challenging. With gastric cancers being the fifth most common cancer around the world and one of the leading causes of cancer-related death^8^, improving quality and safety of cancer surgery is crucial^9^. Globally, the standard of care for curative surgery is gastrectomy with D2 lymphadenectomy, most commonly through a traditional open approach^10^. In recent years, laparoscopic gastrectomy (LG) has gained popularity, following large randomized clinical trials (RCTs) demonstrating non-inferior oncological outcomes compared with open surgery^11–13^. However, the majority of evidence has been from East Asia, resulting in geographical difference in the adoption of LG. In East Asia, minimally invasive approaches accounted for over 70% of gastrectomies in Korea^14^, and similar rates in Japan, by 2019^15^. Meanwhile, LG adoption has been slower in Europe and North America. In Italy, rates of LG increased from 10.8% in 2015 to 26.3% in 2020^16^, and in the Netherland reached 80.6% by 2021^17^; however, in the UK and USA, adoption remains low^18,19^. Gastrectomy with D2 lymphadenectomy remains technically demanding, requiring meticulous dissection and advanced operative skill^20^.

In contrast, robotic-assisted surgery offers technical and ergonomic advantages, and has been postulated to be associated with better clinical outcomes^21^. However, current assessment of the benefits of robotic-assisted gastrectomy (RAG) compared to open or laparoscopic approaches have been limited, due to lack of robust evaluation from published systematic reviews and meta-analyses, including GRADE assessment of certainty^22^. Therefore, the aim of the present study was to evaluate the quality and safety of curative-intent RAG compared to conventional approaches for gastric cancers.

## Methods

### Search strategy and selection criteria

This systematic review and meta-analysis was conducted and reported in accordance with the PRISMA 2020 statement^23^ (*Appendix S1*). The study protocol was registered with PROSPERO (ID: CRD420251034915). An initial systematic search was performed on the MEDLINE (via PubMed), Embase (via Ovid), Web of Science, and Scopus databases from inception to 29 February 2025. The same search was repeated on 2 May 2025 to capture studies published in March and April 2025. No additional studies were included. No publication date restrictions were imposed beyond the final search date. The search strategies used combinations of controlled vocabulary (for example, MeSH) and keywords pertinent to the condition (gastric cancer), intervention (robotic gastrectomy), comparators (laparoscopic gastrectomy, open gastrectomy), and appropriate study designs. The comprehensive search strategy implemented for MEDLINE is detailed in *Appendix S2*. In addition, reference lists of identified studies and relevant systematic reviews were manually scrutinised to identify further eligible studies.

### Inclusion and exclusion criteria

The inclusion criteria for this systematic review were comparative studies (that is, cohort studies or RCTs) including adult (age ≥ 18 years) patients undergoing primary total or subtotal gastrectomy for gastric adenocarcinoma. Eligible studies compared robotic gastrectomy (either totally robotic or robotic-assisted procedures using standardized licensed robotic platforms) with standard of care approaches such as LG or open gastrectomy (OG). The exclusion criteria were: non-comparative studies; inclusion of non-cancer surgery, such as gastrointestinal stromal tumours; and studies evaluating hybrid or hand-assisted surgical procedures.

Titles and abstracts identified through the search were screened by two independent reviewers (RS, WKC) against the predefined eligibility criteria. Full texts of potentially eligible studies were retrieved and assessed independently by the same two reviewers. Disagreements arising at any stage (screening, eligibility assessment, or data extraction) were resolved via discussion and consensus among the reviewers, with arbitration by senior authors (SKK, SRM) implemented when necessary.

Studies excluded after full-text review, along with the reasons for exclusion, are listed in *Appendix S3*.

### Outcome measures

The co-primary outcomes were safety and quality. Safety was assessed based on postoperative complications, defined as the proportion of patients experiencing any complication classified as Clavien–Dindo grade II or higher (CD ≥ II)^24^. Quality was measured according to margin-positive resection (R1), defined according to criteria reported in the included studies. Odds ratios (OR) with their corresponding 95% confidence interval (c.i.) were calculated for these binary outcomes. Studies with zero events in both robotic and conventional arms were included in the forest plots for transparency, but were excluded from the pooled effect estimates.

Secondary outcomes included overall complications (CD ≥ I), major complication (CD ≥ III)^24^, and anastomotic leak. Anastomotic leaks were defined according to criteria reported in the included studies.

### Data extraction

Data extraction was executed independently by two authors (RS, WKC) using a standardized data-extraction form in an Excel (Microsoft, Bellevue, WA, USA) spreadsheet (*Appendix S4*). Two different reviewers (LG, SKK) verified the extracted data for accuracy and completeness. Only summary-level data, as reported in the source publications, were extracted; individual patient data were not sought. To prevent data duplication from overlapping patient cohorts or study periods from the same institution, the most comprehensive or most recent publication presenting unique data was selected as the primary source. Extracted variables were on: study characteristics (that is, industry funding, study design, recruitment period, geographic region); sample size; patient demographics (that is, age, sex, body mass index); tumour characteristics (stage and grade); type of gastrectomy (total, subtotal); and outcome measures such as resection margin status (R0 *versus* R1), postoperative complications, and readmission. Where reported, details of industry funding or support were also extracted.

### Risk of bias

The studies included in this assessment were evaluated for risk of bias using validated, design-specific instruments, namely the Risk Of Bias In Non-randomized Studies of Interventions (ROBINS-I) tool for non-randomized studies^25^ and the Cochrane Risk of Bias tool (RoB 2.0) for RCTs^26^. Two authors (RS, WKC) independently assessed the methodological quality of all the studies with the provided instruments. If consensus was not reached, consensus was reached through discussion with a third independent person (SKK or SRM).

### Statistical analysis

Descriptive data were tabulated to summarize key study characteristics, including sample size, patient demographics, tumour stage distribution, intervention (robotic) and comparator (conventional gastrectomy) groups, outcome definitions, and methodological factors. Both primary and secondary outcomes were binary and analysed by calculating an OR with its 95% c.i. from reported event counts. Where necessary, log(OR) and corresponding standard errors were calculated from raw event data for statistical modelling. Continuous variables were summarized as the mean(standard deviation) or median with interquartile range, as appropriate); categorical variables were summarized as frequencies with percentages. All available studies reporting a given outcome were included in the relevant meta-analysis; missing covariate values were not imputed in meta-regression analyses. Random-effects meta-analyses were performed for each outcome using the restricted maximum likelihood (REML) estimator, which provides a robust approach to estimating between-study variance (τ^2^) when heterogeneity is anticipated. Study-level heterogeneity was quantified using the *I*^2^ statistic and τ^2^; *I*^2^ values of 25, 50, and 75% were interpreted as low, moderate, and high heterogeneity, respectively. Forest plots were generated to visualise study-specific and pooled effect sizes, 95% c.i., study weights, and heterogeneity indices.

Planned meta-regression analyses were conducted to explore potential sources of variation in effect estimates. Prespecified moderators included the proportion of patients with stage III cancer, industry support, the extent of surgical resection (proportion of total gastrectomies), and adoption year, which was defined as the calendar year in which patient recruitment commenced for each study. Studies initiating recruitment before 2015 were classified as early adopters (2015 corresponds to when Intuitive Surgical data demonstrated a sustained increase in global robotic-assisted surgical volume^2,27^). For stage III cancer, an additional exploratory analysis was performed stratifying studies by whether > 50 or ≤ 50% of patients had stage III disease. A subgroup analysis also compared studies from Eastern and Western centres to account for differences in patient populations, disease patterns, and technology adoption. Univariable models were constructed; only studies with complete moderator information were included. Publication bias and small-study effects were assessed for all outcomes using contour-enhanced funnel plots and Egger's regression test for asymmetry. All statistical analyses were conducted in R version 4.3.2 (R Foundation for Statistical Computing, Vienna, Austria) using the meta, metafor, dplyr, and ggplot2 packages.

### Assessing certainty of evidence

The certainty of evidence for each outcome was assessed according to the GRADE approach; GRADEpro^28^ was used to facilitate this process. For each outcome, pooled effect estimates were rated as high, moderate, low, or very low certainty based on five criteria: risk of bias; inconsistency; indirectness; imprecision; and publication bias. Evidence from RCTs was initially classified as high certainty, whereas evidence from non-randomized studies began at low certainty. The certainty rating could be reduced if there were concerns regarding risk of bias, inconsistency, indirectness, imprecision, or small-study effects.

## Results

### Study characteristics

**Fig. 1:**
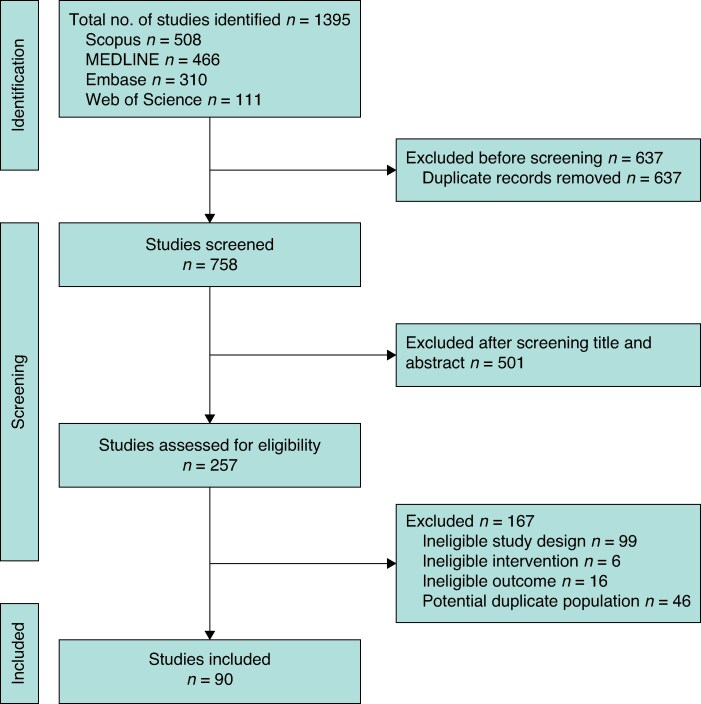
Flow chart of the systematic review

The search identified 1395 articles. After removing duplicates, the titles and abstracts were screened for 758 articles, with 501 articles excluded for not meeting eligibility criteria. This left 257 full-text articles that were assessed for eligibility, of which 167 were excluded. Thus, 90 studies meeting the inclusion criteria were included in the study (*Fig. 1*). Characteristics of the 90^29–118^ included studies are summarized in *Table 1*. The studies encompassed three RCTs and 87 non-randomized studies, with publication dates ranging from 2009 to 2025. The majority of studies originated from China, with 72 studies conducted in East Asia and 17 in Europe and America; 7% of the studies were industry supported.

**Table 1:** Characteristics of studies included in the systematic review

Study	Country	Study period	Design	Industry support	Study groups	No. of patients
Qiao *et al*.^29^ (2025)	China	January 2015–April 2021	Retrospective study	No	RAG	47
					LG	94
Zhang *et al*.^30^ (2024)	China	January 2016–January 2018	Retrospective study	No	RAG	104
					LG	104
Meng *et al*.^31^ (2024)	China	October 2014–September 2022	Retrospective study	No	RAG	79
					LG	79
Nishibeppu *et al*.^32^ (2024)	Japan	November 2018–March 2023	Retrospective study	No	RAG	38
					LG	71
Hondo *et al*.^33^ (2024)	Japan	April 2018–October 2022	Retrospective study	No	RAG	988
					LG	3173
Hwang *et al*.^34^ (2024)	Korea	March 2007–December 2020	Retrospective study	Yes	RAG	147
					LG	204
Wei *et al*.^35^ (2024)	China	August 2016–December 2019	Retrospective study	No	RAG	109
					LG	109
Nishi *et al*.^36^ (2024)	Japan	2007–2023	Retrospective study	No	RAG	48
					LG	42
Wang *et al*.^37^ (2024)	China	NR	Retrospective study	No	RAG	66
					LG	76
Zheng *et al*.^38^ (2024)	China	January 2016–June 2021	Retrospective study	No	RAG	121
					LG	363
Zheng *et al*.^39^ (2024)	China	January 2018–June 2023	Retrospective study	No	RAG	97
					LG	97
Nagata *et al*.^40^ (2024)	Japan	January 2014–December 2022	Retrospective study	No	RAG	73
					LG	73
Kuwabara *et al*.^41^ (2024)	Japan	December 2013–June 2023	Retrospective study	No	RAG	231
					LG	231
Kitazono *et al*.^42^ (2024)	Japan	2016–2020	Retrospective study	No	RAG	32
					LG	32
Kubo *et al*.^43^ (2024)	Japan	January 2015–July 2022	Retrospective study	No	RAG	148
					LG	148
Lu *et al*.^44^ (2024)	China	January 2015–June 2019	Retrospective study	No	RAG	1034
					LG	1034
Hu *et al*.^45^ (2024)	Japan	July 2009–June 2023	Retrospective study	No	RAG	71
					LG	71
Dias *et al*.^46^ (2024)	Brazil	2009–2022	Retrospective study	No	RAG	48
					LG	48
Song *et al*.^47^ (2023)	China	January 2019–October 2021	Retrospective study	No	RAG	135
					LG	155
Trastulli *et al*.^48^ (2023)	International	January 2005–December 2017	Retrospective study	No	RAG	290
					OG	290
Hirata *et al*.^49^ (2023)	USA	January 2018–December 2021	Retrospective study	Yes	RAG	41
					OG	120
Salvador-Rosés *et al*.^50^ (2023)	Spain	2014–2021	Retrospective study	No	RAG	30
					OG	48
Jia *et al*.^51^ (2023)	China	October 2014–October 2021	Retrospective study	No	RAG	147
					LG	371
Tian *et al*.^52^ (2023)	China	February 2020–March 2021	Retrospective study	No	RAG	67
					LG	67
Tian *et al*.^53^ (2023)	China	January 2019–December 2020	Retrospective study	No	RAG	73
					LG	73
Shen *et al*.^54^ (2023)	China	April 2020–October 2021	Retrospective study	No	RAG	67
					LG	46
Takahashi *et al*.^55^ (2023)	USA	2010–2017	Retrospective study	No	RAG	1278
					LG	1278
Suda *et al*.^56^ (2023)	Japan	October 2014–January 2017	Retrospective study	Yes	RAG	326
					LG	757
Li *et al*.^57^ (2023)	China	March 2010–October 2019	Retrospective study	No	RAG	1776
					LG	1776
Miyai *et al*.^58^ (2023)	Japan	April 2015–December 2022	Retrospective study	No	RAG	90
					LG	90
Lee *et al*.^59^ (2023)	Korea	May 2021–May 2022	Prospective study	No	RAG	96
					LG	88
Lin *et al*.^60^ (2023)	China	January 2005–September 2016	Retrospective study	No	RAG	82
					LG	164
Huang *et al*.^61^ (2022)	China	2016–2019	Retrospective study	No	RAG	67
					LG	67
Omori *et al*.^62^ (2022)	Japan	January 2014–December 2019	Retrospective study	No	RAG	210
					LG	210
Kostov *et al*.^63^ (2022)	Bulgaria	January 2018–August 2022	Retrospective study	No	RAG	38
					LG	72
Teranishi *et al*.^64^ (2022)	Japan	2017–2020	Retrospective study	No	RAG	45
					LG	120
Ebihara *et al*.^65^ (2022)	Japan	July 2014–August 2020	Retrospective study	No	RAG	28
					LG	28
Hikage *et al*.^66^ (2022)	Japan	January 2012–December 2020	Retrospective study	No	RAG	394
					LG	882
Gao *et al*.^67^ (2022)	China	January 2015–October 2021	Retrospective study	No	RAG	410
					LG	410
Li *et al*.^68^ (2022)	China	July 2018–February 2022	Retrospective study	No	RAG	16
					LG	110
Li *et al*.^69^ (2022)	China	July 2016–July 2018	Retrospective study	No	RAG	221
					LG	663
Kumamoto *et al*.^70^ (2022)	Japan	June 2017–July 2021	Retrospective study	No	RAG	27
					LG	29
Shibasaki *et al*.^71^ (2022)	Japan	January 2009–June 2021	Retrospective study	No	RAG	118
					LG	193
Yi *et al*.^72^ (2022)	China	September 2016–December 2018	Prospective study	No	RAG	30
					LG	81
Li *et al*.^73^ (2022)	China	March 2018–July 2021	Prospective study	No	RAG	69
					LG	73
Suda *et al*.^74^ (2022)	Japan	October 2018–December 2019	Retrospective study	Yes	RAG	2671
					LG	7671
Kaida *et al*.^75^ (2022)	Japan	January 2011–December 2020	Retrospective study	No	RAG	34
					LG	34
Choi *et al*.^76^ (2021)	Korea	March 2009–June 2018	Retrospective study	Yes	RAG	54
					LG	62
					OG	69
Li *et al*.^77^ (2021)	China	May 2006–October 2019	Retrospective study	No	RAG	29
					LG	41
Garbarino *et al*.^78^ (2021)	Italy	September 2012–March 2017	Retrospective study	No	RAG	43
					OG	43
Ojima *et al*.^79^ (2021)	Japan	April 2018–October 2020	RCT	No	RAG	117
					LG	119
Isobe *et al*.^80^ (2021)	Japan	February 2018–August 2020	Retrospective study	No	RAG	50
					LG	50
Li *et al*.^81^ (2021)	China	March 2010–August 2019	Retrospective study	No	RAG	516
					LG	516
Okabe *et al*.^82^ (2021)	Japan	January 2012–March 2020	Retrospective study	No	RAG	93
					LG	93
Caruso *et al*.^83^ (2020)	Spain	November 2016–February 2019	Prospective study	No	RAG	25
					OG	25
Balbona *et al*.^84^ (2020)	USA	January 2000–May 2018	Retrospective study	No	RAG	46
					OG	198
Aktas *et al*.^85^ (2020)	Turkey	December 2013–March 2018	Retrospective study	No	RAG	30
					LG	64
Wang *et al*.^86^ (2019)	China	January 2016–May 2018	Retrospective study	No	RAG	223
					LG	223
Sun *et al*.^87^ (2019)	China	January 2016–April 2018	Retrospective study	Yes	RAG	33
					LG	88
Kubota *et al*.^88^ (2019)	Japan	February 2015–February 2019	Retrospective study	No	RAG	21
					LG	119
Gao *et al*.^89^ (2019)	China	January 2011–December 2014	Retrospective study	No	RAG	163
					LG	163
Ojima *et al*.^90^ (2019)	Japan	January 2013–December 2017	Retrospective study	No	RAG	20
					LG	639
Solaini *et al*.^91^ (2019)	Italy	June 2008–January 2018	Retrospective study	No	RAG	49
					OG	49
Wang *et al*.^92^ (2019)	China	April 2012–July 2017	Retrospective study	No	RAG	35
					LG	140
Li *et al*.^93^ (2018)	China	August 2013–March 2017	Retrospective study	No	RAG	112
					LG	112
Lu *et al*.^94^ (2018)	China	July 2016–June 2017	Retrospective study	No	RAG	101
					LG	303
Greenleaf *et al*.^95^ (2017)	USA	January 2010–December 2012	Retrospective study	No	RAG	223
					LG	1487
					OG	4717
Pan *et al*.^96^ (2017)	China	January 2015–August 2016	RCT	No	RAG	102
					LG	61
Parisi *et al*.^97^ (2017)	International		Retrospective study	No	RAG	151
					LG	151
					OG	302
Hong *et al*.^98^ (2016)	Korea	October 2008–December 2015	Retrospective study	No	RAG	232
					LG	232
Kim *et al*.^99^ (2016)	Korea	February 2009–September 2011	Retrospective study	No	RAG	87
					LG	288
Park *et al*.^100^ (2016)	Korea	May 2011–December 2012	Prospective study	No	RAG	223
					LG	211
Kim *et al*.^101^ (2016)	Korea	May 2011–December 2012	Prospective study	No	RAG	185
					LG	185
Wang *et al*.^102^ (2016)	China	May 2012–December 2014	RCT	No	RAG	151
					OG	145
Procopiuc *et al*.^103^ (2016)	Romania	January 2004–December 2013	Retrospective study	No	RAG	18
					OG	29
Cianchi *et al*.^104^ (2016)	Italy	June 2008–September 2015	Retrospective study	No	RAG	30
					LG	41
Glenn *et al*.^105^ (2015)	USA	January 2008–December 2013	Retrospective study	No	RAG	223
					LG	789
					OG	8585
Han *et al*.^106^ (2015)	Korea	June 2008–December 2013	Retrospective study	No	RAG	68
					LG	68
Seo *et al*.^107^ (2015)	Korea	June 2004–March 2009	Retrospective study	No	RAG	40
					LG	40
You *et al*.^108^ (2015)	Korea	January 2014–February 2015	Retrospective study	No	RAG	16
					LG	20
					OG	12
Huang *et al*.^109^ (2014)	Taiwan	July 2008–August 2014	Prospective study	No	RAG	72
					LG	73
Noshiro *et al*.^110^ (2014)	Japan	April 2010–November 2012	Prospective study	No	RAG	21
					LG	160
Hyun *et al*.^111^ (2013)	Korea	February 2009–November 2010	Retrospective study	No	RAG	38
					LG	83
					OG	41
Kim *et al*.^112^ (2012)	Korea	January 2005–December 2010	Retrospective study	No	RAG	436
					LG	861
					OG	4542
Huang *et al*.^113^ (2012)	Taiwan	January 2006–02–2012	Retrospective study	No	RAG	39
					LG	64
					OG	586
Son *et al*.^114^ (2012)	Korea	December 2007–December 2011	Retrospective study	No	RAG	21
					LG	42
Caruso *et al*.^115^ (2011)	Italy	January 2005–June 2010	Retrospective study	No	RAG	29
					OG	120
Kim *et al*.^116^ (2010)	Korea	December 2007–June 2008	Retrospective study	No	RAG	16
					LG	11
					OG	12
Pugliese *et al*.^117^ (2010)	Italy	June 2000–October 2009	Retrospective study	No	RAG	18
					LG	52
Song *et al*.^118^ (2009)	Korea	May 2003–November 2006	Retrospective study	No	RAG	20
					LG	40

RAG, robotic-assisted gastrectomy; LG, laparoscopic gastrectomy; NR, not reported; OG, open gastrectomy; RCT, randomized clinical trial.

### Patient characteristics

In all, 65 296 patients were included across all studies, with 16 580 undergoing RAG, 28 783 undergoing LG, and 19 933 undergoing OG. The weighted mean proportion of patients undergoing total gastrectomy was 39%, whereas the pooled proportion of patients with pathological stage III disease was 32% across all studies. The characteristics of patients in the included studies are summarized in *Table 2*.

**Table 2:** Characteristics of patients in the included studies

Study	Groups	No. of patients	Age (years)	Sex		Gastrectomy		Cancer stages	Stage 3	BMI (kg/m^2^)
				Male	Female	Total	Subtotal			
Qiao *et al*.^29^ (2025)	RAG	47	40	24 (51%)	23 (49%)	NR	NR	Early and advanced	44 (31%)	22.2
	LG	94	40	60 (64%)	34 (36%)					22.8
Zhang *et al*.^30^ (2024)	RAG	104	56.9	63 (61%)	41 (39%)	13 (6%)	195 (94%)	Early and advanced	31 (15%)	21.7
	LG	104	57.3	59 (57%)	45 (43%)					21.6
Meng *et al*.^31^ (2024)	RAG	79	63.6	64 (81%)	15 (19%)	119 (75%)	39 (25%)	Early and advanced	40 (25%)	31.6
	LG	79	63.6	55 (70%)	24 (30%)					31.4
Nishibeppu *et al*.^32^ (2024)	RAG	38	NR	NR	NR	9 (8%)	100 (92%)	Early and advanced	8 (7%)	NR
	LG	71								
Hondo *et al*.^33^ (2024)	RAG	988	NR	632 (64%)	356 (36%)	NR	NR	Early and advanced	NR	NR
	LG	3173		2023 (64%)	1150 (36%)					
Hwang *et al*.^34^ (2024)	RAG	147	53.1	78 (53%)	69 (47%)	351 (100%)		Advanced	133 (38%)	23
	LG	204	59.3	136 (67%)	68 (33%)					23.5
Wei *et al*.^35^ (2024)	RAG	109	63	83 (76%)	26 (24%)	110 (50%)	108 (50%)	Early and advanced	73 (33%)	26.75
	LG	109	61	78 (72%)	31 (28%)					26.75
Nishi *et al*.^36^ (2024)	RAG	48	72	30 (83%)	6 (17%)	NR	NR	Early and advanced	39 (43%)	22.9
	LG	42	67.5	17 (53%)	15 (47%)					22.4
Wang *et al*.^37^ (2024)	RAG	66	58.6	48 (73%)	18 (27%)	NR	NR	Early and advanced	48 (34%)	NR
	LG	76	60	50 (66%)	26 (34%)					
Zheng *et al*.^38^ (2024)	RAG	121	69	98 (81%)	23 (19%)	292 (60%)	192 (40%)	Advanced	227 (47%)	23.6
	LG	363	69.7	270 (74%)	93 (26%)					23.4
Zheng Y *et al*.^39^ (2024)	RAG	97	57.9	66 (68%)	31 (32%)	99 (44%)	128 (56%)	Early and advanced	78 (40%)	23
	LG	97	58.6	67 (69%)	30 (31%)					23
Nagata *et al*.^40^ (2024)	RAG	73	68	48 (66%)	25 (34%)	146 (100%)		Early and advanced	36 (25%)	21.9
	LG	73	68	50 (68%)	23 (32%)					22.4
Kuwabara *et al*.^41^ (2024)	RAG	231	71	150 (65%)	81 (35%)	69 (15%)	393 (85%)	Early and advanced	91 (24%)	22.8
	LG	231	69	145 (63%)	86 (37%)					22.7
Kitazono *et al*.^42^ (2024)	RAG	32	67.6	21 (66%)	11 (34%)		64 (100%)	Early and advanced	5 (8%)	23.7
	LG	32	69.1	23 (72%)	9 (28%)					23.4
Kubo *et al*.^43^ (2024)	RAG	148	67.7	96 (65%)	52 (35%)	32 (11%)	261 (89%)	Early and advanced	49 (16%)	21
	LG	148	66.8	94 (64%)	54 (36%)					20.9
Lu *et al*.^44^ (2024)	RAG	1034	59.9	731 (71%)	303 (29%)	644 (31%)	1424 (69%)	Early and advanced	791 (38%)	22.2
	LG	1034	59.8	742 (72%)	292 (28%)					22.3
Hu *et al*.^45^ (2024)	RAG	71	68	54 (76%)	17 (24%)		142 (100%)	Early and advanced	NR	23.1
	LG	71	67	60 (85%)	11 (15%)					23.5
Dias *et al*.^46^ (2024)	RAG	48	58.3	25 (52%)	23 (48%)	27 (28%)	69 (72%)	Early and advanced	34 (35%)	23.7
	LG	48	53	26 (54%)	22 (46%)					23.9
Song *et al*.^47^ (2023)	RAG	135	63.8	83 (72%)	32 (28%)		290 (100%)	Early and advanced	117 (4%)	24.1
	LG	155	65.5	95 (61%)	60 (39%)					24
Trastulli *et al*.^48^ (2023)	RAG	290	68.7	165 (57%)	125 (43%)	216 (37%)	364 (63%)	Early and advanced	176 (3%)	24.1
	OG	290	68.1	172 (59%)	118 (41%)					24.2
Hirata *et al*.^49^ (2023)	RAG	41	60	24 (59%)	17 (41%)	83 (52%)	78 (48%)	Early and advanced	114 (71%)	25
	OG	120	64	82 (68%)	38 (32%)					26
Salvador-Rosés *et al*.^50^ (2023)	RAG	30	68	23 (77%)	7 (23%)	78 (100%)		Early and advanced	52 (67%)	26
	OG	48	64	40 (83%)	8 (17%)					27
Jia *et al*.^51^ (2023)	RAG	147	62.9	118 (80%)	29 (20%)	NR	NR	Early and advanced	220 (42%)	24.9
	LG	371	62.5	294 (79%)	77 (21%)					24.5
Tian *et al*.^52^ (2023)	RAG	67	59.2	45 (67%)	22 (33%)	NR	NR	Advanced	79 (59%)	24.1
	LG	67	57.4	47 (70%)	20 (30%)					25.4
Tian *et al*.^53^ (2023)	RAG	73	55.9	49 (67%)	24 (33%)	NR	NR	Early and advanced	30 (21%)	23.8
	LG	73	54.8	46 (63%)	27 (37%)					23.8
Shen *et al*.^54^ (2023)	RAG	67	65.7	43 (64%)	24 (36%)	NR	NR	Early and advanced	45 (40%)	22.9
	LG	46	64.2	25 (54%)	21 (46%)					22.1
Takahashi *et al*.^55^ (2023)	RAG	1278	65	914 (71%)	364 (29%)	236 (18%)	1082 (82%)	Early and advanced	650 (25%)	NR
	LG	1278	65	899 (70%)	379 (30%)					
Suda *et al*.^56^ (2023)	RAG	326	66	201 (62%)	125 (38%)	231 (21%)	852 (79%)	Early	NR	22.4
	LG	757	68	506 (67%)	251 (33%)					22.3
Li *et al*.^57^ (2023)	RAG	1776	57.6	1276 (72%)	500 (28%)	994 (28%)	2558 (72%)	Early and advanced	1894 (53%)	22.5
	LG	1776	57.8	1279 (72%)	497 (28%)					22.4
Miyai *et al*.^58^ (2023)	RAG	90	70.2	62 (69%)	28 (31%)	56 (31%)	124 (69%)	Early and advanced	63 (35%)	22.5
	LG	90	70.1	65 (72%)	25 (28%)					22.6
Lee *et al*.^59^ (2023)	RAG	96	61.8	57 (59%)	39 (41%)	31 (17%)	153 (83%)	Early and advanced	36 (20%)	24
	LG	88	64.3	53 (60%)	35 (40%)					23.9
Lin *et al*.^60^ (2023)	RAG	82	61.6	69 (84%)	13 (16%)	NR	NR	Early and advanced	160 (65%)	22.7
	LG	164	61.6	138 (84%)	26 (16%)					22.6
Huang *et al*.^61^ (2022)	RAG	67	49.3	43 (64%)	24 (36%)	NR	NR	Early and advanced	41 (31%)	22.5
	LG	67	49.1	47 (70%)	20 (30%)					22.3
Omori *et al*.^62^ (2022)	RAG	210	66	152 (72%)	58 (28%)	80 (19%)	340 (81%)	Early and advanced	104 (25%)	22.8
	LG	210	65.5	153 (73%)	57 (27%)					22.7
Kostov *et al*.^63^ (2022)	RAG	38	63.1	24 (63%)	14 (37%)	NR	NR	Early and advanced	59 (54%)	23.6
	LG	72	62.1	33 (46%)	39 (54%)					23.9
Teranishi *et al*.^64^ (2022)	RAG	45	69	31 (69%)	14 (31%)	NR	NR	Early and advanced	NR	22.5
	LG	120	72	82 (68%)	38 (32%)					22.4
Ebihara *et al*.^65^ (2022)	RAG	28	72.5	17 (61%)	11 (39%)	NR	NR	Early and advanced	2 (4%)	22.1
	LG	28	71	17 (61%)	11 (39%)					22.2
Hikage *et al*.^66^ (2022)	RAG	394	68	251 (64%)	143 (36%)	96 (8%)	1180 (92%)	Early	NR	21.3
	LG	882	70	620 (70%)	262 (30%)					21.1
Gao *et al*.^67^ (2022)	RAG	410	59.8	284 (69%)	126 (31%)		820 (100%)	Early and advanced	483 (59%)	23.1
	LG	410	59.7	301 (73%)	109 (27%)					23.1
Li *et al*.^68^ (2022)	RAG	16	54.4	10 (63%)	6 (38%)	52 (41%)	74 (59%)	Early and advanced	22 (17%)	24.14
	LG	110	56.9	77 (70%)	33 (30%)					23.1
Li *et al*.^69^ (2022)	RAG	221	58.4	165 (75%)	56 (25%)	482 (55%)	402 (45%)	Early and advanced	382 (43%)	23
	LG	663	58.7	509 (77%)	154 (23%)					22.9
Kumamoto *et al*.^70^ (2022)	RAG	27	69	19 (70%)	8 (30%)	NR	NR	Early and advanced	20 (36%)	23.2
	LG	29	70	19 (66%)	10 (34%)					22.4
Shibasaki *et al*.^71^ (2022)	RAG	118	69	69 (69%)	31 (31%)	NR	NR	Early and advanced	NR	23
	LG	193	68	67 (67%)	33 (33%)					23.1
Yi *et al*.^72^ (2022)	RAG	30	57.8	18 (60%)	12 (40%)	NR	NR	Early and advanced	82 (74%)	23.6
	LG	81	58.3	48 (59%)	33 (41%)					23.9
Li *et al*.^73^ (2022)	RAG	69	59.36	48 (70%)	21 (30%)	NR	NR	Early and advanced	77 (54%)	22.6
	LG	73	58.9	52 (71%)	21 (29%)					22.8
Suda *et al*.^74^ (2022)	RAG	2671	NR	1760 (66%)	911 (34%)	776 (25%)	4566 (85%)	NR	1131 (11%)	NR
	LG	7671		1754 (66%)	917 (34%)					
Kaida *et al*.^75^ (2022)	RAG	34	69	19 (56%)	15 (44%)	19 (28%)	49 (72%)	Early	NR	23.1
	LG	34	69	18 (53%)	16 (47%)					23.2
Choi *et al*.^76^ (2021)	RAG	54	59	40 (74%)	14 (26%)	33 (23%)	109 (77%)	Early and advanced	59 (32%)	26.5
	LG	62	63	45 (73%)	17 (27%)					26.6
	OG	69	66	47 (68%)	22 (32%)					26.5
Li *et al*.^77^ (2021)	RAG	29	60.3	22 (76%)	7 (24%)		70 (100%)	Early and advanced	43 (61%)	19.4
	LG	41	58.2	31 (76%)	10 (24%)					20.4
Garbarino *et al*.^78^ (2021)	RAG	43	77.7	23 (53%)	20 (47%)	21 (24%)	65 (76%)	Early and advanced	39 (45%)	23.3
	OG	43	78.5	22 (51%)	21 (49%)					23.8
Ojima *et al*.^79^ (2021)	RAG	117	71	73 (62%)	44 (38%)	58 (25%)	172 (75%)	Early and advanced	45 (19%)	22.4
	LG	119	72	77 (65%)	42 (35%)					21.9
Isobe *et al*.^80^ (2021)	RAG	50	69.2	31 (62%)	19 (38%)		100 (100%)	Early and advanced	13 (13%)	23
	LG	50	69.3	34 (68%)	16 (32%)					22.9
Li *et al*.^81^ (2021)	RAG	516	55.1	354 (69%)	162 (31%)		1032 (100%)	Early and advanced	342 (33%)	NR
	LG	516	54.6	333 (65%)	183 (35%)					
Okabe *et al*.^82^ (2021)	RAG	93	69	62 (67%)	31 (33%)	60 (32%)	126 (68%)	Early and advanced	78 (42%)	23.6
	LG	93	70	57 (61%)	36 (39%)					22.9
Caruso *et al*.^83^ (2020)	RAG	25	64	NR	NR	50 (100%)		Early and advanced	19 (38%)	21.8
	OG	25	68.7							22
Balbona *et al*.^84^ (2020)	RAG	46	61.9	26 (57%)	20 (43%)	45 (40%)	67 (60%)	Early and advanced	55 (23%)	NR
	OG	198	65.5	65 (42%)	91 (58%)					
Aktas *et al*.^85^ (2020)	RAG	30	55	18 (60%)	12 (40%)	50 (43%)	44 (47%)	Early and advanced	51 (54%)	26
	LG	64	59	41 (64%)	23 (36%)					24
Wang *et al*.^86^ (2019)	RAG	223	57.7	183 (81%)	43 (19%)	193 (43%)	253 (57%)	Early and advanced	172 (39%)	22.1
	LG	223	57.4	180 (81%)	43 (19%)					22.2
Sun *et al*.^87^ (2019)	RAG	33	55.6	24 (73%)	9 (27%)	NR	NR	Early and advanced	22 (18%)	22.38
	LG	88	54.7	65 (74%)	23 (26%)					22.5
Kubota *et al*.^88^ (2019)	RAG	21	59.8	11 (52%)	10 (48%)	NR	NR	Early and advanced	NR	20.9
	LG	119	67	72 (61%)	47 (39%)					22.3
Gao *et al*.^89^ (2019)	RAG	163	60.27	121 (74%)	42 (26%)	122 (37%)	204 (63%)	Early and advanced	206 (63%)	23.7
	LG	163	59.8	125 (77%)	38 (23%)					23.3
Ojima *et al*.^90^ (2019)	RAG	20	71	13 (65%)	7 (35%)	NR	NR	Early and advanced	89 (19%)	21.5
	LG	639	70	430 (67%)	209 (33%)					22
Solaini *et al*.^91^ (2019)	RAG	49	NR	29 (59%)	20 (41%)		98 (100%)	Early and advanced	68 (69%)	24.2
	OG	49		28 (57%)	21 (43%)					24.8
Wang *et al*.^92^ (2019)	RAG	35	55.3	29 (83%)	6 (17%)	175 (100%)		Early and advanced	91 (52%)	23
	LG	140	55.1	123 (88%)	17 (12%)					23.1
Li *et al*.^93^ (2018)	RAG	112	55.6	78 (70%)	34 (30%)	NR	NR	Early and advanced	98 (44%)	23.6
	LG	112	56.1	79 (71%)	33 (29%)					23.6
Lu *et al*.^94^ (2018)	RAG	101	NR	73 (72%)	28 (28%)	170 (42%)	234 (58%)	Early and advanced	154 (38%)	NR
	LG	303		212 (70%)	91 (30%)					
Greenleaf *et al*.^95^ (2017)	RAG	223	63.7	157 (70%)	66 (30%)	2187 (35%)	4048 (65%)	Early and advanced	3351 (52%)	NR
	LG	1487	63.9	1038 (70%)	449 (30%)					
	OG	4717	63.7	3245 (69%)	1472 (31%)					
Pan *et al*.^96^ (2017)	RAG	102	65.13	65 (64%)	37 (36%)	98 (60%)	65 (40%)	Early and advanced	48 (29%)	24.12
	LG	61	65.6	45 (74%)	16 (26%)					23.9
Parisi *et al*.^97^ (2017)	RAG	151	68.81	81 (54%)	70 (46%)	186 (31%)	418 (69%)	Early and advanced	152 (25%)	24.58
	LG	151	65.8	85 (56%)	66 (44%)					24
	OG	302	67.2	185 (61%)	117 (39%)					24.3
Hong *et al*.^98^ (2016)	RAG	232	53.7	154 (66%)	78 (34%)	NR	NR	Early and advanced	40 (9%)	23.8
	LG	232	55	156 (67%)	76 (33%)					23.8
Kim *et al*.^99^ (2016)	RAG	87	54.1	46 (53%)	41 (47%)	NR	NR	Early and advanced	3 (1%)	21.1
	LG	288	60.5	170 (59%)	118 (41%)					24
Park *et al*.^100^ (2016)	RAG	223	52.6	131(59%)	92 (41%)	72 (17%)	362 (83%)	Early and advanced	NR	NR
	LG	211	55.8	126 (60%)	85 (40%)					
Kim *et al*.^101^ (2016)	RAG	185	53.3	113 (61%)	72 (39%)	60 (16%)	310 (84%)	Early and advanced	16 (4%)	23.8
	LG	185	56	113 (61%)	72 (39%)					23.6
Wang *et al*.^102^ (2016)	RAG	151	57.5	109 (72%)	42 (28%)	NR	NR	Early and advanced	143 (48%)	22.1
	OG	145	55.9	89 (61%)	56 (39%)					21.3
Procopiuc *et al*.^103^ (2016)	RAG	18	59.17	13 (72%)	5 (28%)	33 (70%)	14 (30%)	Advanced	23 (49%)	26.5
	OG	29	60.1	21 (72%)	8 (28%)					24.8
Cianchi *et al*.^104^ (2016)	RAG	30	73	14 (47%)	16 (53%)		71(100%)	Early and advanced	20 (28%)	27
	LG	41	74	19 (46%)	22 (54%)					26
Glenn *et al*.^105^ (2015)	RAG	223	NR	153 (69%)	70 (31%)	NR	NR	NR	NR	NR
	LG	789		499 (63%)	290 (37%)					
	OG	8585		5899 (69%)	2686 (31%)					
Han *et al*.^106^ (2015)	RAG	68	50.6	31 (46%)	37 (54%)	136 (100%)		Early and advanced	1 (1%)	22.7
	LG	68	49.8	32 (47%)	36 (53%)					22.8
Seo *et al*.^107^ (2015)	RAG	40	51.6	19 (48%)	21 (53%)		80 (100%)	Early and advanced	2 (3%)	23.6
	LG	40	55.1	20 (50%)	20 (50%)					23.8
You *et al*.^108^ (2015)	RAG	16	57.7	10 (63%)	6 (38%)	NR	NR	Early and advanced	5 (1%)	23
	LG	20	67.1	14 (70%)	6 (30%)					22.8
	OG	12	61.8	8 (67%)	4 (33%)					25.4
Huang *et al*.^109^ (2014)	RAG	72	67.7	40 (56%)	32 (44%)	18 (12%)	127 (88%)	Early and advanced	12 (8%)	24.1
	LG	73	66	42 (58%)	31 (42%)					24.2
Noshiro *et al*.^110^ (2014)	RAG	21	66	14 (67%)	7 (33%)		181 (100%)	Early and advanced	NR	22.8
	LG	160	69	102 (64%)	58 (36%)					21.8
Hyun *et al*.^111^ (2013)	RAG	38	54.2	25 (66%)	13 (34%)	NR	NR	Early and advanced	10 (6%)	23.8
	LG	83	60.3	55 (66%)	28 (34%)					23.8
	OG	41	57.7	28 (68%)	13 (32%)					22.7
Kim *et al*.^112^ (2012)	RAG	436	54.2	266 (61%)	171(39%)	1499 (26%)	4339 (74%)	Early and advanced	1388 (24%)	23.6
	LG	861	58.8	550 (64%)	311(36%)					23.5
	OG	4542	57.7	3008 (66%)	1534 (34%)					23.3
Huang *et al*.^113^ (2012)	RAG	39	65.1	19 (49%)	20 (51%)	193 (28%)	496 (72%)	Early and advanced	285 (41%)	24.2
	LG	64	65.6	43 (67%)	21 (33%)					24.7
	OG	586	67.9	406 (69%)	180 (31%)					23.7
Son *et al*.^114^ (2012)	RAG	21	52.3	14 (67%)	7 (33%)	3 (4%)	69 (96%)	Early and advanced	9 (14%)	23.7
	LG	42	52.8	26 (76%)	8 (24%)					22.6
Caruso *et al*.^115^ (2011)	RAG	29	64.8	18 (62%)	11 (38%)	49 (33%)	100 (67%)	Early and advanced	37 (25%)	27
	OG	120	65.1	65 (54%)	55 (46%)					28
Kim *et al*.^116^ (2010)	RAG	16	53.8	10 (63%)	6 (38%)		27 (100%)	Early and advanced	3 (8%)	21.3
	LG	11	57.9	10 (91%)	1 (9%)					25.3
	OG	12	56	9 (75%)	3 (25%)					25.2
Pugliese *et al*.^117^ (2010)	RAG	18	NR	7 (39%)	11 (61%)		70 (100%)	Early and advanced	NR	NR
	LG	52		35 (67%)	17 (33%)					
Song *et al*.^118^ (2009)	RAG	20	51.6	8 (48%)	12 (60%)		60 (100%)	Early	NR	23.4
	LG	40	55	27 (68%)	13 (33%)					23

^*^Values are *n* (%) unless otherwise stated.Age and BMI are presented as the mean; reported as mean or median depending on the study. BMI, body mass index; RAG, robotic-assisted gastrectomy; LG, laparoscopic gastrectomy; NR, not reported; OG, open gastrectomy.

### Safety of robotic-assisted surgery

**Fig. 2:**
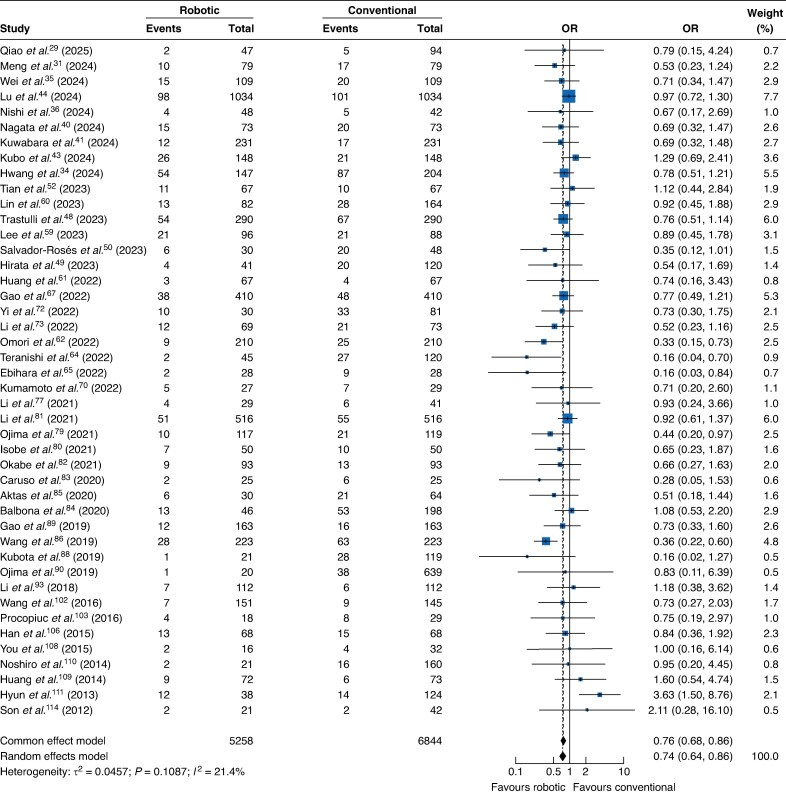
Forest plot showing a random-effects meta-analysis of safety, measured by Clavien–Dindo grade ≥ II complications, comparing robotic-assisted and conventional gastrectomy Values in parentheses are 95% confidence intervals. OR, odds ratio.

The co-primary outcome for safety was postoperative complications, defined as CD ≥ II. Data were available from 44 studies (2 RCTs), encompassing 12 102 patients and 911 events. RAG was associated with significantly lower odds of complications compared with conventional approaches (OR 0.74; 95% c.i. 0.64 to 0.86; *Fig. 2*). Between-study heterogeneity was low (*I*^2^=21.4%; τ^2^=0.045; *P* = 0.10). Seven studies were assessed as low risk of bias. Meta-regression was performed to explore sources of heterogeneity using four study-level covariates. None was significantly associated with the effect estimates for CD ≥ II. A subgroup analysis by geographic region confirmed this consistency, showing a benefit for RAG in both Eastern (37 studies; OR 0.75; 95% c.i. 0.64 to 0.89) and Western (7 studies; OR 0.69; 95% c.i. 0.52 to 0.94) cohorts, with no significant subgroup difference (*P* = 0.65; *Fig. S1*). Funnel plot inspection did not reveal considerable asymmetry, and Egger’s linear regression test was non-significant (*t* = −1.42; d.f. = 42; *P* = 0.16), suggesting a low likelihood of small-study effects, including potential publication bias (*Fig. S2*). The certainty of evidence was rated as very low according to GRADE criteria (*Table 3*).

**Table 3:** GRADE summary table showing certainty of evidence for Clavien–Dindo grade ≥ II complications and margin-positive resection

Certainty assessment							No. of patients		Effect*		Certainty
No. of studies	Study design	Risk of bias	Inconsistency	Indirectness	Imprecision	Other considerations	Robotic gastrectomy	Conventional gastrectomy†	Relative	Absolute	
**Clavien–Dindo grade ≥ II complication**											
44	Non-randomized studies	Serious‡	Not serious	Not serious	Not serious	All plausible residual confounding would reduce the demonstrated effect	324 of 5258 (6.2%)	587 of 6844 (8.6%)	OR 0.74 (0.64 to 0.86)	21 fewer per 1000 (from 29 fewer to 11 fewer)	⨁◯◯◯ Very low‡
**Positive resection margin**											
35	Non-randomized studies	Serious‡	Not serious	Not serious	Serious§	All plausible residual confounding would reduce the demonstrated effect	156 of 8459 (18.5%)	712 of 25 861 (2.8%)	OR 0.74 (0.51 to 1.07)	7 fewer per 1000 (from 13 fewer to 2 more)	⨁◯◯◯ Very low‡,§

^*^Values in parentheses are 95% confidence intervals. †Laparoscopic and open gastrectomy. ‡Non-randomized studies with majority high or serious risk of bias. §Imprecision in estimates. OR, odds ratio.

### Quality of robotic-assisted surgery

**Fig. 3:**
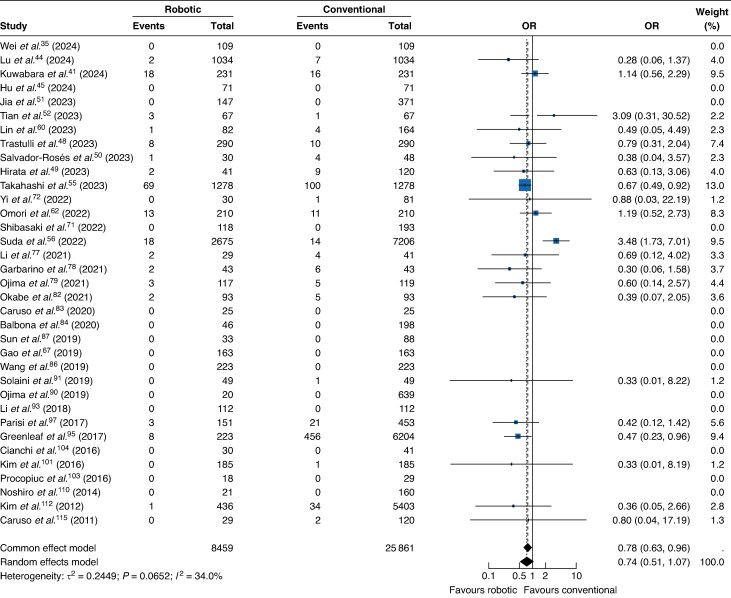
Forest plot showing a random-effects meta-analysis of quality, measured by positive margin resection (R1), comparing robotic-assisted and conventional gastrectomy Values in parentheses are 95% confidence intervals. OR, odds ratio.

Margin status was reported in 35 studies, comprising 34 320 patients and 868 events. RAG was associated with lower odds of R1 resection compared with conventional approaches (OR 0.74; 95% c.i. 0.51 to 1.07; *Fig. 3*), although the difference was not statistically significant. Between-study heterogeneity was moderate (*I*^2^ = 34.0%; τ^2^ = 0.24; *P* = 0.06). Three studies were assessed as low risk of bias. Meta-regression revealed that early adoption had a borderline correlation with effect size (*P* = 0.0635), accounting for 41.1% of heterogeneity. Subgroup analysis demonstrated lower rates of R1 resection for RAG performed before 2015 (*P* = 0.0017), but no change in later studies when compared to conventional approaches (*P* = 0.83; *Fig. S3*). Meta-regression also demonstrated that industry funding was strongly linked to more favourable effect estimates (*P* < 0.0001) and fully explained the observed heterogeneity. Subgroup analysis demonstrated a significant effect in non-industry-supported studies (*P* = 0.0005), but non-significant effects and marked heterogeneity among industry-supported studies (*P* = 0.51; *Fig. S4*). Robotic surgery was associated with lower rates of R1 resection in studies with ≥ 50% stage III patients (*P* = 0.05) and accounted for 49.4% of heterogeneity between studies (*Fig. S5*). A complementary subgroup analysis comparing studies with > 50 and ≤ 50% stage III patients supported this finding, with a clearer treatment effect observed in groups with a higher proportion of patients with stage III cancer (*Fig. S6*). Subgroup analysis by region revealed no significant subgroup difference (*P* = 0.18). In Eastern studies (12 studies), RAG was not associated with R1 rates (OR 0.94; 95% c.i. 0.54 to 1.64), whereas in Western studies (9 studies), RAG was associated with significantly lower R1 rates (OR 0.62; 95% c.i. 0.47 to 0.80; *Fig. S7*). Funnel plot inspection did not reveal considerable asymmetry, and Egger’s linear regression test was non-significant (*t* = −0.73; d.f. = 19; *P* = 0.47), suggesting a low likelihood of small-study effects, including potential publication bias (*Fig. S8*). The certainty of evidence was rated as very low according to GRADE criteria (*Table 3*).

### Secondary outcome measures

Data on major complications (CD ≥ III) were available from 58 studies, encompassing 36 952 patients and 1519 events. RAG was associated with significantly lower odds of major complications compared with conventional approaches (OR 0.74; 95% c.i. 0.60 to 0.90; *Fig. S9*). Heterogeneity was substantial (*I*^2^ = 65.1%; τ^2^ = 0.21; *P* < 0.0001). Meta-regression revealed that industry support was significantly associated with more favourable effect estimates in favour of RAG (*P* = 0.0013), accounting for 39.3% of the between-study heterogeneity (*Fig. S10*). Subgroup analysis confirmed a statistically significant benefit of RAG among non-industry-supported studies (*P* < 0.0001; *I*^2^ = 0%). In contrast, among industry-supported studies, effect estimates were not statistically significant (*P* = 0.46) and showed substantial heterogeneity (*I*^2^ = 84.6%).

Data on anastomotic leak were available from 69 studies, including 41 444 patients and 687 events. There was no significant difference in the odds of anastomotic leak between robotic and conventional gastrectomy (OR 1.06; 95% c.i. 0.90 to 1.25; *Fig. S11*). Heterogeneity across studies was low (*I*^2^ = 8.8%; τ^2^ = 0.0136; *P* = 0.27).

Data on overall complications were available from 44 studies, encompassing 33 245 patients. RG was associated with significantly lower odds of overall complications compared with conventional approaches (OR 0.83; 95% c.i. 0.73 to 0.94; *Fig. S12*). Heterogeneity was moderate (*I*^2^ = 30.6%; τ^2^ = 0.0422; *P* = 0.0319). This heterogeneity was not explained by a multivariable meta-regression because no included study-level covariates were significantly associated with the effect estimate.

## Discussion

This systematic review and meta-analysis provides evidence around the safety and quality of RAG compared with conventional OG or LG. Evidence from 90 studies and over 65 000 patients demonstrates that robotic surgery is associated with lower postoperative complications and a trend towards fewer R1 resections. However, the direction of these estimates was influenced by studies with industry involvement or early adoption. Taken together, the certainty of evidence according to GRADE for quality and safety is very low. This emphasises an urgent need for better evaluation of current robotic-assisted platforms for gastrectomies and other surgeries, before the widespread scaling of these technologies across health systems.

This study represents the most comprehensive and methodologically rigorous synthesis of the available evidence to date. Although other systematic reviews and meta-analyses have previously assessed robotic surgery for gastric cancer^21–28,119–129^, the majority have been limited by methodological shortcomings, including inadequate handling of heterogeneity, lack of systematic risk-of-bias assessments, and the absence of GRADE evaluation. In contrast, this review used comprehensive analyses and meta-regression techniques to systematically explore heterogeneity, ensuring a greater reliability and robustness of the findings. Moreover, this is one of the first systematic reviews to explicitly address potential sources of bias associated with industry involvement, providing a more balanced interpretation of clinical outcomes compared with previous publications.

RAG has demonstrated several technical advantages, likely underpinning its improved outcomes compared with conventional approaches. The enhanced visualization provided by high-definition, three-dimensional imaging enables superior anatomical delineation and precise lymphadenectomy, potentially reducing surgical trauma^130^. The robotic platform’s increased dexterity, instrument stability, and tremor filtration also facilitate meticulous dissection and suturing, particularly in technically challenging areas such as the splenic hilum and perigastric vessels^131^. These technical refinements may explain observed reductions in postoperative complications and potentially improved margin status. Furthermore, ergonomically superior robotic platforms may minimize surgeon fatigue, indirectly contributing to consistently higher surgical quality^132^. Despite overall benefit, considerable heterogeneity was observed across the included studies. Meta-regression analyses identified key contributors to this variability. Notably, temporal trends had a significant effect: studies conducted in earlier periods tended to report more favourable outcomes for robotic gastrectomy, particularly regarding R1 resection rates. This pattern likely reflects more selective patient inclusion in the early adoption phase, rather than learning curve effects alone. Furthermore, R1 interpretation also depends heavily on the margin's specific location^133^, a detail unavailable in the aggregated data from the included studies. As robotic techniques became more widely adopted and indications broadened, the apparent benefits diminished, suggesting that changes in patient selection criteria over time played a major role in outcome heterogeneity.

A particularly salient finding of this review was the significant association between industry sponsorship and more favourable outcomes for RAG, which explained a substantial portion of heterogeneity for both R1 resection rates and major complications. However, the 7% of studies declaring direct industry support likely represents only the tip of the iceberg. Undeclared financial conflicts of interest are prevalent in the surgical literature^134,135^, and they extend beyond direct research funding to include consulting fees, speaker honoraria, and paid proctorship roles. In the context of robotic surgery, proctoring by experienced surgeons is a key component of training, yet these proctors are often paid independent contractors, with their services coordinated by the device manufacturer^136^. Such financial relationships are frequently not disclosed as a conflict of interest within subsequent research publications, yet they have been shown to correlate with the publication of pro-industry findings^137^. This unmeasured confounding from undeclared financial ties may have significantly biased the existing evidence base in favour of the robotic platform.

### Implications for practice and policy

The findings of this review have important implications for health system leaders, surgical policymakers, and clinicians considering the adoption or scale-up of robotic platforms for gastric cancer surgery. The results indicate that robotic-assisted gastrectomy is associated with lower rates of postoperative complications and a trend towards improved oncological quality indicators, suggesting that, when performed in appropriate settings, robotic surgery can enhance the safety and technical quality of complex gastrointestinal cancer operations. These benefits, if realised consistently across health systems, have the potential to translate into downstream improvements in patient recovery, reductions in postoperative morbidity, shorter hospital stays, and lower rates of readmission, each of which carries implications for hospital efficiency and cost containment. However, these potential advantages must be carefully balanced against the substantial capital and maintenance costs associated with robotic systems^138,139^. In publicly funded health systems such as the UK NHS, where surgical innovation competes for limited resources, clear evidence of clinical and economic benefit is essential to justify investment. This review adds important clinical context to recent health technology assessments, such as those led by NICE, which have highlighted the need for robust evidence not only on comparative safety and effectiveness but also on cost-effectiveness and learning curve requirements. Centralization of complex oncological procedures to high-volume centres with demonstrated outcomes may also be warranted to optimize clinical benefit and resource utilization^140^. Equity in access must also be a central consideration. At present, robotic systems are more likely to be deployed in tertiary centres and affluent regions, raising the risk of widening disparities in access to high-quality surgical care^141,142^. Policymakers must consider mechanisms such as national procurement strategies, coordinated workforce training, and outcome-based funding models that promote equitable access while maintaining high standards of care^143^. Lastly, the integration of robotic surgery into cancer care pathways should be embedded within ongoing quality assurance frameworks and prospective registries. This would enable real-time monitoring of outcomes, benchmarking of institutional performance, and iterative learning across the system, aligning innovation with accountability and continuous improvement.

### Implications for research

Future research should address the key evidence gaps that remain following this review. First, high-quality, multicentre RCTs directly comparing RAG and LG are needed, with rigorous surgical quality assurance, long-term oncological outcomes, and stratification by tumour stage and procedure type. Second, robust health economic evaluations across a range of health systems, particularly in low- and middle-income countries, are essential to determine the context-specific value and affordability of robotic platforms, incorporating capital, maintenance, and training costs alongside clinical benefits. Third, future studies should expand beyond traditional clinical endpoints to include patient-reported outcomes^144^, such as quality of life, postoperative functional recovery, and return to work, as well as assessments of surgical ergonomics^145^ and team dynamics, to ensure a holistic understanding of the impact of robotic surgery in routine clinical practice.

### Strengths and limitations

The major strength of this systematic review and meta-analysis is the methodological robustness in its assessment of 90 studies encompassing over 65 000 patients, enabling precise effect estimates across a broad range of populations and practice settings. This review adhered to rigorous methodological standards throughout, in accordance with the GRADE framework, to assess the certainty of evidence for each outcome, strengthening the interpretability and clinical utility of the findings. The present analysis went beyond simple effect estimation by using a random-effects model that explicitly accounted for anticipated clinical and methodological heterogeneity. Importantly, extensive meta-regression and subgroup analyses were conducted to explore sources of heterogeneity, including industry support, disease stage, patient characteristics, and temporal effect. These efforts enabled plausible sources of variability to be identified and contextual interpretation of the results to be provided.

Despite these strengths, several limitations must be acknowledged. Most notably, the body of evidence is dominated by non-randomized observational studies, which are inherently susceptible to confounding, selection bias, and reporting bias. Although robust methodological tools were used to assess and mitigate these risks, residual confounding from unmeasured variables, such as surgeon experience, institutional volume, and perioperative protocols, remains a possibility and may have influenced the observed associations. Second, although RAG was associated with improved outcomes, these benefits may partly reflect centre- or surgeon-level expertise rather than the intrinsic superiority of the robotic platform itself. High-volume, technologically advanced centres are more likely to adopt robotic systems, and outcomes in such centres may not be generalizable to lower-volume or resource-constrained settings^146,147^. Furthermore, a small number of studies declared industry funding or support, which may have introduced sponsorship bias. In addition, by design, the analysis combined laparoscopic and open procedures into a single ‘conventional approach’ comparator to reflect current standard of care. A limitation of this approach is that it cannot distinguish whether the observed benefits of robotic surgery are more pronounced against open or laparoscopic techniques. Third, substantial variation in outcome definitions, reporting standards, and complication grading systems limited data harmonization across studies. For example, definitions of anastomotic leak were inconsistently reported, and follow-up durations varied, precluding reliable analysis of long-term outcomes such as disease-free or overall survival. In addition, the meta-regression addressed the mix of total and subtotal gastrectomies at a study level, but a key limitation remains the pooling of these clinically distinct procedures. Furthermore, the data extraction protocol did not include the use of neoadjuvant chemotherapy and, as such, its role as a potential confounder could not be directly assessed. This lack of standardization may have introduced outcome misclassification and contributed to between-study heterogeneity. To maintain a focused scope, this review deliberately excluded outcomes such as lymph node yield, operative time, and medium-term metrics (for example, readmission and reoperation rates). Fourth, although declared industry support was identified as a significant source of heterogeneity, it was not possible to fully account for undisclosed financial or non-financial conflicts of interest. Given the widespread prevalence of such undisclosed relationships among surgeons, as documented by systems like Open Payments^134^, the true influence of industry likely exceeds what the present analysis captured, introducing substantial unmeasured confounding. Thus, the findings of this study highlight the critical need for structured, independent training and credentialing programs. As previously discussed, industry-led training is inherently conflicted due to its primary goal of promoting platform adoption rather than objectively assessing competency. To safeguard patient safety and quality, training and assessment programs should instead be designed and governed independently by professional surgical societies and healthcare institutions, emphasizing proficiency-based progression and ongoing quality assurance.

Finally, although evaluation of learning curve effects and temporal trends was attempted, individual-level data were not available to rigorously model surgeon or institutional learning trajectories. Similarly, cost data were variably reported or absent, precluding formal assessment of cost-effectiveness, which is essential for health policy decision-making, particularly in publicly funded health systems.

## Supplementary Material

zraf126_Supplementary_Data

## Data Availability

Data sharing requests will be considered by the writing group upon written request to the corresponding author.
