# Supplementary material for: Evaluating safety and quality of robotic-assisted gastric cancer surgery: meta-analysis and meta-regression
Source: BJS Open. 2025 Nov 24;9(6):zraf126. doi: 10.1093/bjsopen/zraf126 (PMC12641133; doi:10.1093/bjsopen/zraf126)

**Evaluating safety and quality of robotic-assisted gastric cancer surgery globally: A systematic review, meta-analysis and meta-regression**

Riadh Salem^1^, Wing Chou^1^, Lorenzo Giorgi^1^, Sivesh K. Kamarajah ^*2^, Sheraz R. Markar ^*1,3^

*Joint senior authors

1. Nuffield Department of Surgical Sciences, University of Oxford, Oxford
2. Department of Applied Health Sciences, University of Birmingham, Birmingham
3. Department of Surgery, Churchill Hospital, Oxford University Hospitals NHS Trust, Oxford, UK.

**Corresponding Author:** Professor Sheraz R Markar PhD (Imperial), PhD (Karolinska), FRCS, Surgical Intervention Trials Unit, Nuffield Department of Surgical Sciences, University of Oxford, Oxford, United Kingdom. Email: [sheraz.markar@nds.ox.ac.uk](mailto:sheraz.markar@nds.ox.ac.uk)

**Number of words:** 2951 **Number of pages:** 28

**Number of tables:** 4

**Number of figures:** 16

**Key words:** robotic surgical procedures, gastrectomy, stomach neoplasms, quality assurance, patient safety

**Short title:** Safety and quality of robotic-assisted gastric cancer surgery

**Data sharing:** Data sharing requests will be considered by the writing group upon written request to the corresponding authors.

**Conflicts of interest:** There are no conflicts of interest to declare.

Table of Contents

[Appendix S1: PRISMA 2020 statement checklist 3](#_Toc210414049)

[Appendix S2: Search strategy for MEDLINE 6](#_Toc210414050)

[Appendix S3: List of studies excluded at full-text screening stage 7](#_Toc210414051)

[Appendix S4: Data extraction sheet 11](#_Toc210414052)

[Appendix S5: Supplementary Figures for Primary Outcomes 12](#_Toc210414053)

[Appendix S6: Secondary outcomes 22](#_Toc210414054)

[Clavien-Dindo grade III 22](#_Toc210414055)

[Anastomotic leak 24](#_Toc210414056)

[Overall Complication 25](#_Toc210414057)

[GRADE for secondary outcomes 26](#_Toc210414058)

[Appendix S7: ROBINS-I and ROB2 Risk of Bias 27](#_Toc210414059)

## Appendix S1: PRISMA 2020 statement checklist

*Table S1 PRISMA 2020 statement checklist*

| **Section and Topic** | **Item #** | **Checklist item** | **Location where item is reported** |
| --- | --- | --- | --- |
| **TITLE** | | |  |
| Title | 1 | Identify the report as a systematic review. | 1 |
| **ABSTRACT** | | |  |
| Abstract | 2 | See the PRISMA 2020 for Abstracts checklist. | 2-3 |
| **INTRODUCTION** | | |  |
| Rationale | 3 | Describe the rationale for the review in the context of existing knowledge. | 4-5 |
| Objectives | 4 | Provide an explicit statement of the objective(s) or question(s) the review addresses. | 4-5 |
| **METHODS** | | |  |
| Eligibility criteria | 5 | Specify the inclusion and exclusion criteria for the review and how studies were grouped for the syntheses. | 6 |
| Information sources | 6 | Specify all databases, registers, websites, organisations, reference lists and other sources searched or consulted to identify studies. Specify the date when each source was last searched or consulted. | 6 |
| Search strategy | 7 | Present the full search strategies for all databases, registers and websites, including any filters and limits used. | 6 |
| Selection process | 8 | Specify the methods used to decide whether a study met the inclusion criteria of the review, including how many reviewers screened each record and each report retrieved, whether they worked independently, and if applicable, details of automation tools used in the process. | 6 |
| Data collection process | 9 | Specify the methods used to collect data from reports, including how many reviewers collected data from each report, whether they worked independently, any processes for obtaining or confirming data from study investigators, and if applicable, details of automation tools used in the process. | 7 |
| Data items | 10a | List and define all outcomes for which data were sought. Specify whether all results that were compatible with each outcome domain in each study were sought (e.g. for all measures, time points, analyses), and if not, the methods used to decide which results to collect. | 6-7 |
|  | 10b | List and define all other variables for which data were sought (e.g. participant and intervention characteristics, funding sources). Describe any assumptions made about any missing or unclear information. | 6-7 |
| Study risk of bias assessment | 11 | Specify the methods used to assess risk of bias in the included studies, including details of the tool(s) used, how many reviewers assessed each study and whether they worked independently, and if applicable, details of automation tools used in the process. | 7 |
| Effect measures | 12 | Specify for each outcome the effect measure(s) (e.g. risk ratio, mean difference) used in the synthesis or presentation of results. | 7-8 |
| Synthesis methods | 13a | Describe the processes used to decide which studies were eligible for each synthesis (e.g. tabulating the study intervention characteristics and comparing against the planned groups for each synthesis (item #5)). | 7-8 |
|  | 13b | Describe any methods required to prepare the data for presentation or synthesis, such as handling of missing summary statistics, or data conversions. | 7-8 |
|  | 13c | Describe any methods used to tabulate or visually display results of individual studies and syntheses. | 7 |
|  | 13d | Describe any methods used to synthesize results and provide a rationale for the choice(s). If meta-analysis was performed, describe the model(s), method(s) to identify the presence and extent of statistical heterogeneity, and software package(s) used. | 7-8 |
|  | 13e | Describe any methods used to explore possible causes of heterogeneity among study results (e.g. subgroup analysis, meta-regression). | 8 |
|  | 13f | Describe any sensitivity analyses conducted to assess robustness of the synthesized results. |  |
| Reporting bias assessment | 14 | Describe any methods used to assess risk of bias due to missing results in a synthesis (arising from reporting biases). | 7 |
| Certainty assessment | 15 | Describe any methods used to assess certainty (or confidence) in the body of evidence for an outcome. | 8 |
| **RESULTS** | | |  |
| Study selection | 16a | Describe the results of the search and selection process, from the number of records identified in the search to the number of studies included in the review, ideally using a flow diagram. | 10 |
|  | 16b | Cite studies that might appear to meet the inclusion criteria, but which were excluded, and explain why they were excluded. | Appendix |
| Study characteristics | 17 | Cite each included study and present its characteristics. | 25-31 |
| Risk of bias in studies | 18 | Present assessments of risk of bias for each included study. | Appendix |
| Results of individual studies | 19 | For all outcomes, present, for each study: (a) summary statistics for each group (where appropriate) and (b) an effect estimate and its precision (e.g. confidence/credible interval), ideally using structured tables or plots. | 10-11, 39-40 |
| Results of syntheses | 20a | For each synthesis, briefly summarise the characteristics and risk of bias among contributing studies. | 10-11 |
|  | 20b | Present results of all statistical syntheses conducted. If meta-analysis was done, present for each the summary estimate and its precision (e.g. confidence/credible interval) and measures of statistical heterogeneity. If comparing groups, describe the direction of the effect. | 10-11, 39-40 |
|  | 20c | Present results of all investigations of possible causes of heterogeneity among study results. | 10-11, Appendix |
|  | 20d | Present results of all sensitivity analyses conducted to assess the robustness of the synthesized results. |  |
| Reporting biases | 21 | Present assessments of risk of bias due to missing results (arising from reporting biases) for each synthesis assessed. | Appendix |
| Certainty of evidence | 22 | Present assessments of certainty (or confidence) in the body of evidence for each outcome assessed. | 37 |
| **DISCUSSION** | | |  |
| Discussion | 23a | Provide a general interpretation of the results in the context of other evidence. | 12-14 |
|  | 23b | Discuss any limitations of the evidence included in the review. | 14-15 |
|  | 23c | Discuss any limitations of the review processes used. | 14-15 |
|  | 23d | Discuss implications of the results for practice, policy, and future research. | 15-16 |
| **OTHER INFORMATION** | | |  |
| Registration and protocol | 24a | Provide registration information for the review, including register name and registration number, or state that the review was not registered. | 2 |
|  | 24b | Indicate where the review protocol can be accessed, or state that a protocol was not prepared. | 12-14 |
|  | 24c | Describe and explain any amendments to information provided at registration or in the protocol. | 6 |
| Support | 25 | Describe sources of financial or non-financial support for the review, and the role of the funders or sponsors in the review. | 1 |
| Competing interests | 26 | Declare any competing interests of review authors. | 1 |
| Availability of data, code and other materials | 27 | Report which of the following are publicly available and where they can be found: template data collection forms; data extracted from included studies; data used for all analyses; analytic code; any other materials used in the review. | 1 |

## Appendix S2: Search strategy for MEDLINE

*Table S2 Search strategy for MEDLINE*

| Set | Advanced Search | Results |
| --- | --- | --- |
| 1 | ("Gastrectomy"[Mesh] OR gastrectomy OR "gastric surgery" OR "stomach surgery" OR "gastric resection" OR "stomach resection") | 69,701 |
| 2 | ("Robotic Surgical Procedures"[Mesh] OR "robotic surgery" OR "robot‑assisted surgery"   OR "robotic gastrectomy" OR "robot‑assisted gastrectomy"   OR "da Vinci" OR hugo OR Versius OR CMR OR intuitive) | 93,995 |
| 3 | (("Laparoscopy"[Mesh] OR "Minimally Invasive Surgical Procedures"[Mesh]     OR laparoscopy OR "laparoscopic surgery" OR "minimally invasive surgery"     OR "laparoscopic gastrectomy" OR "minimally invasive gastrectomy")    OR    ("open surgery" OR "open gastrectomy" OR "conventional surgery" OR "traditional surgery")) | 693,474 |
| 4 | ("Stomach Neoplasms"[Mesh] OR "gastric cancer" OR "stomach cancer" OR "gastric carcinoma"   OR "stomach carcinoma" OR "gastric neoplasm" OR "stomach neoplasm"   OR "gastric adenocarcinoma" OR "stomach adenocarcinoma") | 152,271 |
| 5 | 1 AND 2 | 1203 |
| 6 | 5 AND 3 | 878 |
| 7 | 6 AND 4 | 609 |
| 8 | 7 AND English [lang] AND Humans [Mesh] | 466 |

## Appendix S3: List of studies excluded at full-text screening stage

Table S3 List of excluded studies along with reason for exclusion, note: reviews and meta-analysis are not included.

| Author | Year | DOI | Reason for exclusion |
| --- | --- | --- | --- |
| Kalavacherla | 2025 | 10.1007/s12029-024-01163-y | Ineligible study design |
| Yamamoto | 2025 | 10.1002/ags3.12842 | Ineligible study design |
| Matsui | 2025 | 10.1007/s12029-025-01205-z | Ineligible study design |
| Tanaka | 2024 | 10.1186/s12876-024-03155-5 | Ineligible study design |
| Ye | 2024 | 10.1186/s12957-024-03484-5 | Ineligible study design |
| Ricciardi | 2024 | 10.1097/SLA.0000000000006572 | Ineligible study design |
| Zhong | 2024 | 10.1097/JS9.0000000000001325 | Ineligible study outcomes |
| Li | 2024 | 10.1093/bjs/znad435 | Ineligible study outcomes |
| Akimoto | 2024 | 10.1007/s00464-024-10857-0 | Ineligible study intervention |
| Lu | 2024 | 10.1038/s41467-024-49013-6 | Ineligible study outcomes |
| Araruna | 2024 | 10.1002/jso.27904 | Ineligible study design |
| Cui | 2024 | 10.1093/gastro/goae005 | Ineligible study intervention |
| Gurau | 2024 | 10.1016/j.jss.2024.10.014 | Ineligible study outcomes |
| Hirata | 2023 | 10.14740/wjon1657 | Ineligible study design |
| Jeong | 2023 | 10.3390/cancers15225371 | Ineligible study intervention |
| Liang | 2023 | 10.1007/s00464-023-10125-7 | Ineligible study outcomes |
| Ross | 2023 | 10.1177/00031348231175139 | Ineligible study outcomes |
| Shimada | 2023 | 10.1245/s10434-023-1332-7 | Ineligible study design |
| Lu | 2023 | 10.1007/s00464-023-10147-1 | Ineligible study outcomes |
| Nishi | 2023 | 10.1186/s12893-023-02045-z | Ineligible study design |
| Hikage | 2022 | 10.1007/s00464-021-08903-2 | Potential of duplicate data |
| Hirata | 2022 | 10.1245/s10434-022-11836-2 | Ineligible study outcomes |
| Kubo | 2022 | 10.1245/s10434-022-11410-w | Ineligible study outcomes |
| Kamarajah | 2022 | 10.1245/s10434-021-11082-y | Ineligible study design |
| Li | 2022 | 10.1177/15533506211047011 | Ineligible study intervention |
| Osaki | 2022 | 10.1111/ases.13037 | Ineligible study design |
| Yoshikawa | 2022 | 10.2152/jmi.69.261 | Ineligible study design |
| Hikage | 2021 | 10.1007/s00268-020-05939-8 | Potential of duplicate data |
| Hikage | 2021 | 10.1007/s00464-020-08224-w | Potential of duplicate data |
| Nakauchi | 2021 | 10.1245/s10434-021-09798-y | Ineligible study design |
| Hikage | 2021 | 10.1007/s00464-020-07895-9 | Potential of duplicate data |
| Li | 2021 | 10.1007/s00464-020-08198-9 | Ineligible study outcomes |
| Schneider | 2021 | 10.1007/s00464-020-08123-0 | Ineligible study outcomes |
| Lu | 2020 | 10.1245/s10434-019-08170-5 | Ineligible study outcomes |
| Li | 2020 | 10.1002/rcs.2148 | Ineligible study design |
| Matsunaga | 2020 | 10.33160/yam.2020.05.005 | Ineligible study outcomes |
| Roh | 2020 | 10.1038/s41598-020-57413-z | Ineligible study intervention |
| Shibasaki | 2020 | 10.3748/wjg.v26.i11.1172 | Ineligible study design |
| Luo | 2019 | 10.1186/s12957-019-1722-5 | Ineligible study intervention |
| Pak | 2019 | 10.1016/j.jgo.2018.10.012 | Ineligible study design |
| Sweigert | 2019 | 10.1016/j.surg.2019.05.041 | Ineligible study design |
| Uyama | 2019 | 10.1007/s10120-018-00906-8 | Ineligible study design |
| Ye | 2019 | 10.1186/s12893-019-0549-x | Ineligible study outcomes |
| Li | 2019 | 10.3892/ijo.2019.4851 | Ineligible study design |
| Li | 2018 | 10.1007/s00464-017-5826-0 | Ineligible study outcomes |
| Hikage | 2018 | 10.1007/s00268-017-4345-4 | Potential of duplicate data |
| Park | 2012 | 10.1002/bjs.8887 | Ineligible study outcomes |
| Pugliese | 2009 | 10.1016/j.ejso.2008.02.001 | Potential of duplicate data |
| Lin | 2024 | 10.1007/s00464-024-10769-z | Potential of duplicate data |
| Fu | 2023 | 10.3389/fsurg.2022.1057496 | Potential of duplicate data |
| Maegawa | 2023 | 10.1007/s00464-023-10519-7 | Potential of duplicate data |
| Komatsu | 2023 | 10.1007/s00595-023-02681-1 | Potential of duplicate data |
| Shigeno | 2023 | 10.1016/j.surg.2022.12.022 | Potential of duplicate data |
| Ribeiro | 2022 | 10.1007/s11605-022-05448-0 | Potential of duplicate data |
| Kinoshita | 2022 | 10.1007/s00464-021-08483-1 | Potential of duplicate data |
| Perez Holguin | 2022 | 10.1016/j.jss.2022.05.017 | Potential of duplicate data |
| Tian | 2022 | 10.1007/s00464-020-08253-5 | Potential of duplicate data |
| Wang | 2022 | 10.1007/s00464-022-09352-1 | Potential of duplicate data |
| Chen | 2022 | 10.1097/SLA.0000000000004764 | Potential of duplicate data |
| Shin | 2021 | 10.1097/SLA.0000000000003845 | Potential of duplicate data |
| Roh | 2021 | 10.1038/s41598-021-95017-3 | Potential of duplicate data |
| Lu | 2021 | 10.1097/SLA.0000000000004466 | Potential of duplicate data |
| Yang | 2020 | 10.1186/s12885-020-07160-1 | Potential of duplicate data |
| Song | 2020 | 10.5230/jgc.2020.20.e36 | Potential of duplicate data |
| Ryan | 2020 | 10.1177/1553350619868113 | Potential of duplicate data |
| Ye | 2020 | 10.1038/s41598-020-63616-1 | Potential of duplicate data |
| Alhossaini | 2020 | 10.1007/s00464-019-06838-3 | Potential of duplicate data |
| Kong | 2020 | 10.1007/s11605-019-04158-4 | Potential of duplicate data |
| Seo | 2020 | 10.1038/s41598-020-73118-9 | Potential of duplicate data |
| Caruso | 2019 | 10.1007/s13304-018-0533-5 | Potential of duplicate data |
| Zhang | 2018 | 10.1177/1073274818765999 | Potential of duplicate data |
| Liu | 2018 | 10.1016/j.ijsu.2018.05.015 | Potential of duplicate data |
| Obama | 2018 | 10.1007/s10120-017-0740-7 | Potential of duplicate data |
| Yang | 2017 | 10.1245/s10434-017-5851-1 | Potential of duplicate data |
| Nakauchi | 2016 | 10.1007/s00464-016-4904-z | Potential of duplicate data |
| Shen | 2016 | 10.1007/s00464-015-4241-7 | Potential of duplicate data |
| Kikuchi | 2016 | 10.1111/ases.12288 | Potential of duplicate data |
| Park | 2015 | 10.1007/s00268-015-2998-4 | Potential of duplicate data |
| Suda | 2015 | 10.1007/s00464-014-3718-0 | Potential of duplicate data |
| Lee | 2015 | 10.1007/s00464-015-4069-1 | Potential of duplicate data |
| Junfeng | 2014 | 10.1007/s00464-013-3385-6 | Potential of duplicate data |
| Kim | 2014 | 10.1016/j.ejso.2013.09.011 | Potential of duplicate data |
| Son | 2014 | 10.1007/s00464-014-3511-0 | Potential of duplicate data |
| Uyama | 2012 | 10.1007/s00268-011-1352-8 | Potential of duplicate data |
| Eom | 2012 | 10.1016/j.ejso.2011.09.006 | Potential of duplicate data |
| Yoon | 2012 | 10.1007/s00464-011-2043-0 | Potential of duplicate data |
| Kang | 2012 | 10.5230/jgc.2012.12.3.156 | Potential of duplicate data |
| Woo | 2011 | 10.1001/archsurg.2011.114 | Potential of duplicate data |

## Appendix S4: Data extraction sheet

Table S4 Extraction sheet table, listing the main variables and key outcomes collected.

| **Category** | **Definition** |
| --- | --- |
| **Study characteristics** | |
| Study name | Includes author name and year |
| Study year | Study starts and end date |
| Adoption year | Defined as the calendar year in which patient recruitment commenced for each study. |
| Country | This is defined as the countries in which the study data was collected. |
| Industry support | Declared financial or material support from commercial industry. |
| **Patient characteristics** | |
| Age | This refers to the mean or median age of the cohort in the study. |
| Sex | Male or Female |
| Body mass index, kg/m2 | The mean or median body max index of patients in the study. |
| Type of Resection | This was either total or subtotal gastrectomy. |
| Ratio of Stage 3 disease | Proportion of patients with Stage 3 disease per TNM classification. |
| **Key outcomes** | |
| Safety | Defined as complications occurring within 90 days post-surgery, classified using the Clavien-Dindo system; anastomotic leaks, as defined by each included study, |
| Quality | Oncological quality was measured by the rate of R1 resection. The definition of R1 resection was based on each individual study’s criteria. |

## Appendix S5: Supplementary Figures for Primary Outcomes

Figure S1 Forest plot of a subgroup analysis, stratified by geographical region showing odds ratios (ORs) with 95% confidence intervals (CIs) for each study comparing robotic (experimental arm) and conventional (control arm) gastrectomy. CD2 Complications.


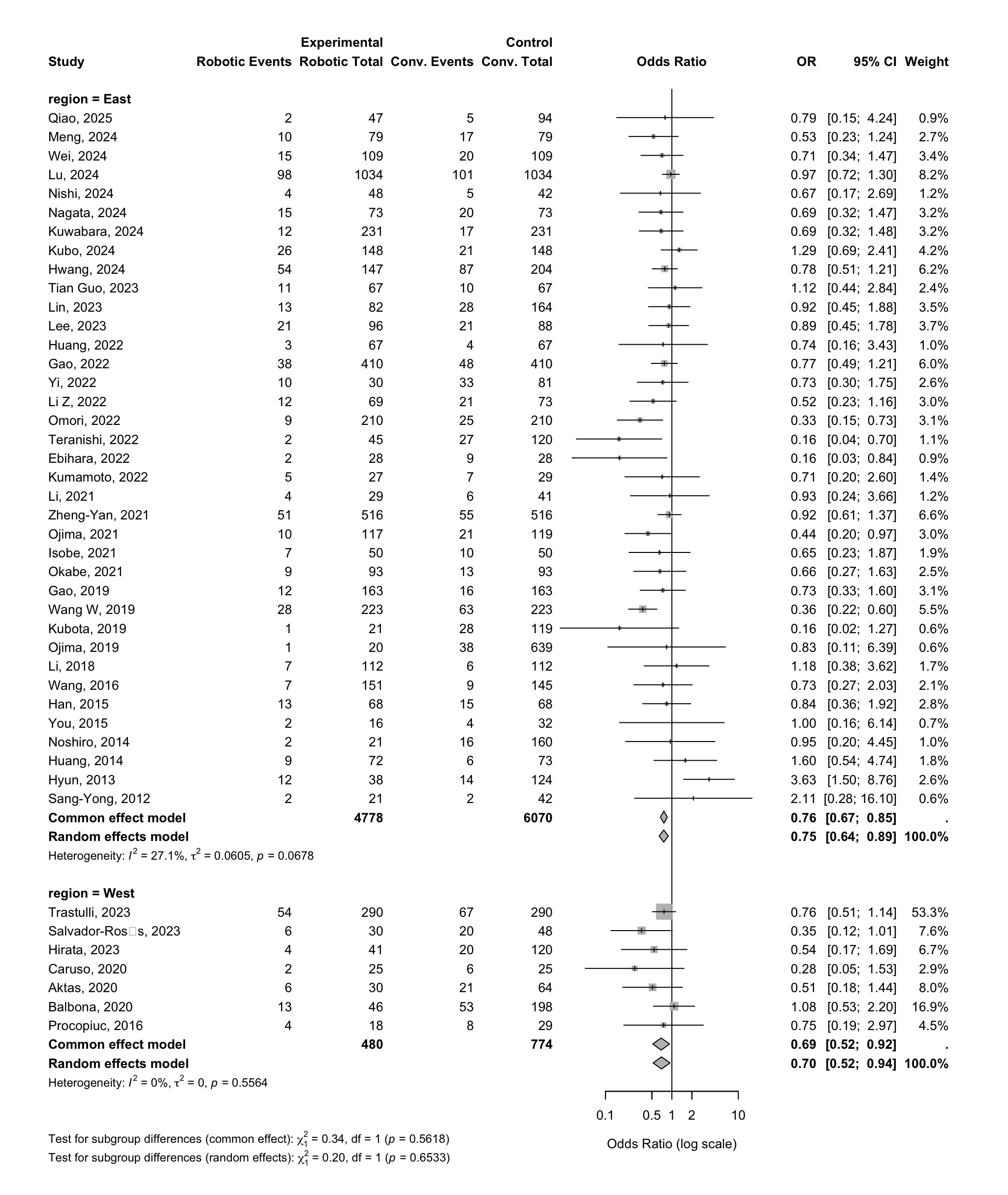


Figure S2-A Funnel plot of studies reporting CD ≥ II complications comparing robotic vs conventional gastrectomy. No substantial asymmetry was observed.


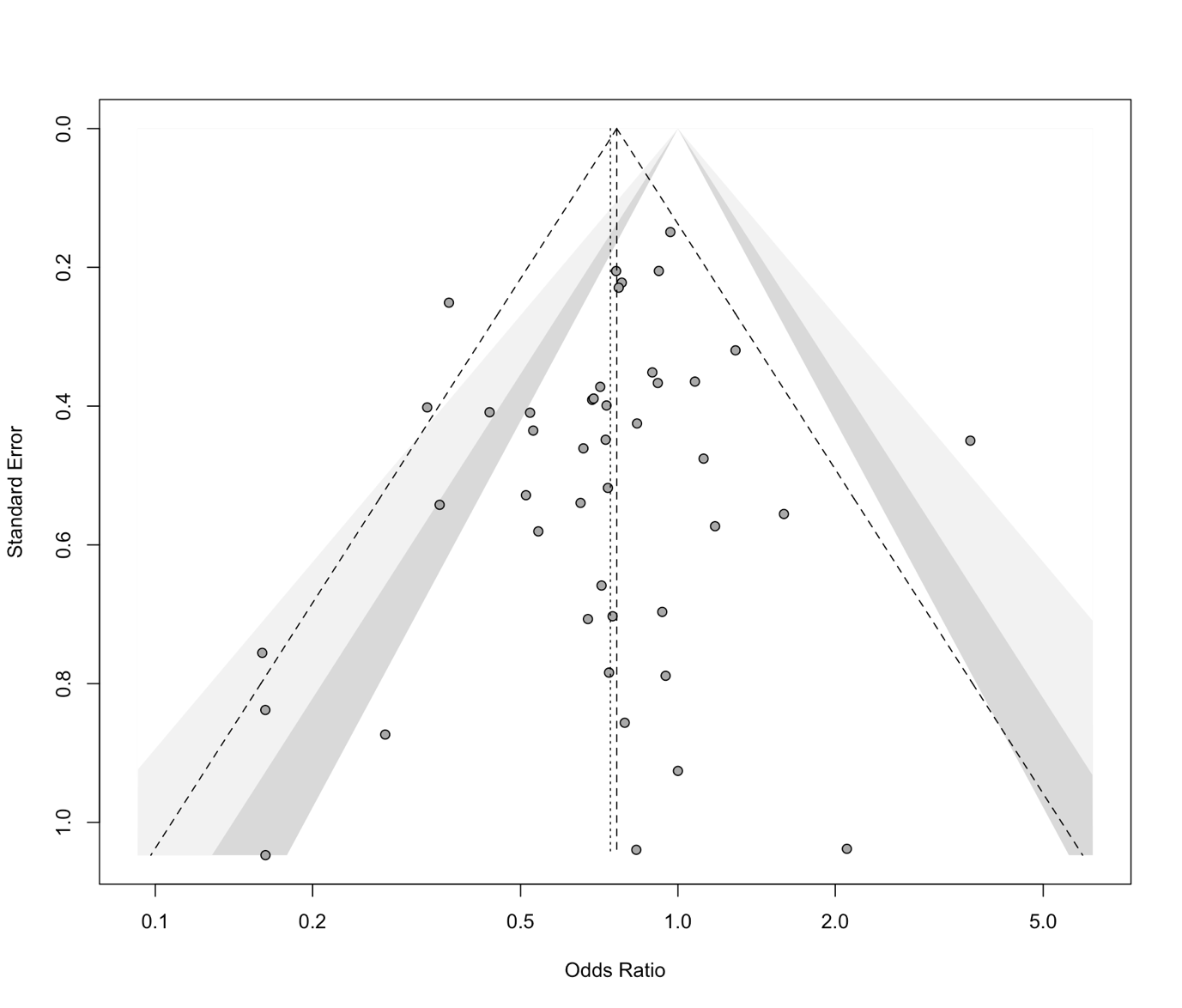


Figure S2-B Egger’s test for CD ≥ II funnel plot asymmetry was non-significant (t = –1.42, df = 42, p = 0.1624), suggesting a low likelihood of small-study effects.


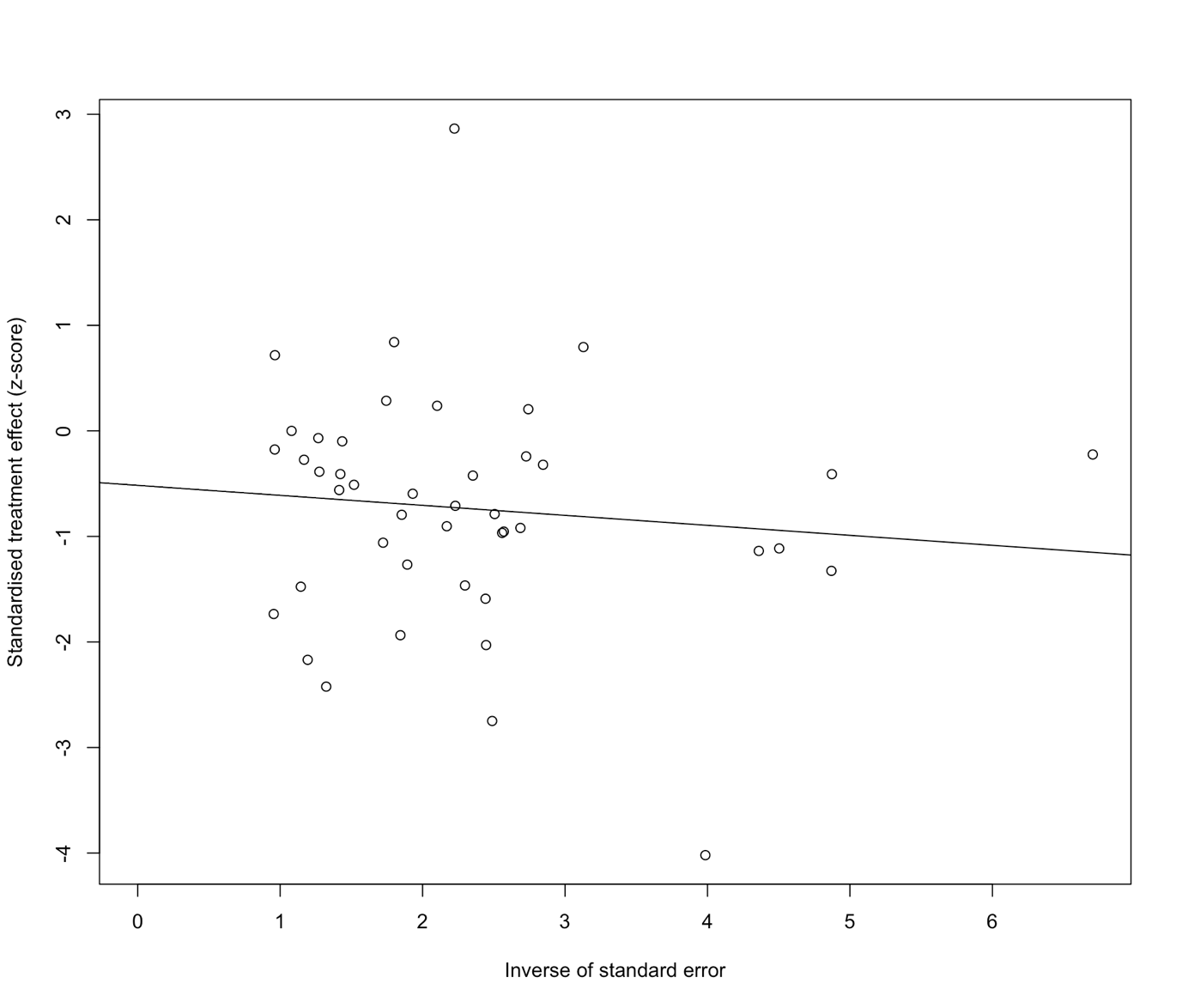


Figure S3 Forest plot of a subgroup analysis, stratified by recruitment period for R1 resection, showing odds ratios (ORs) with 95% confidence intervals (CIs) for each study comparing robotic (experimental arm) and conventional (control arm) gastrectomy. Early = before 2015, Late = after 2015.


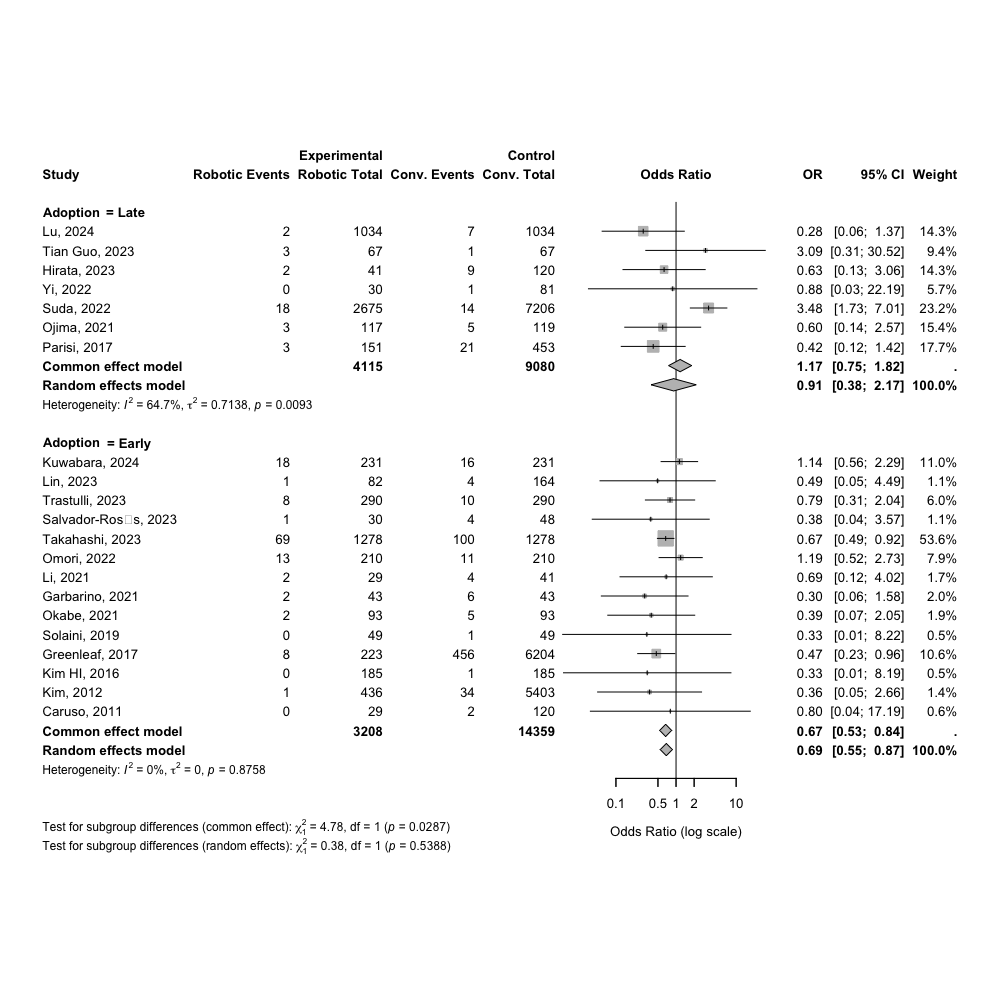


Figure S4 Forest plot of a subgroup analysis, stratified by industry support, for R1 resection, showing odds ratios (ORs) with 95% confidence intervals (CIs) for each study comparing robotic (experimental arm) and conventional (control arm) gastrectomy.


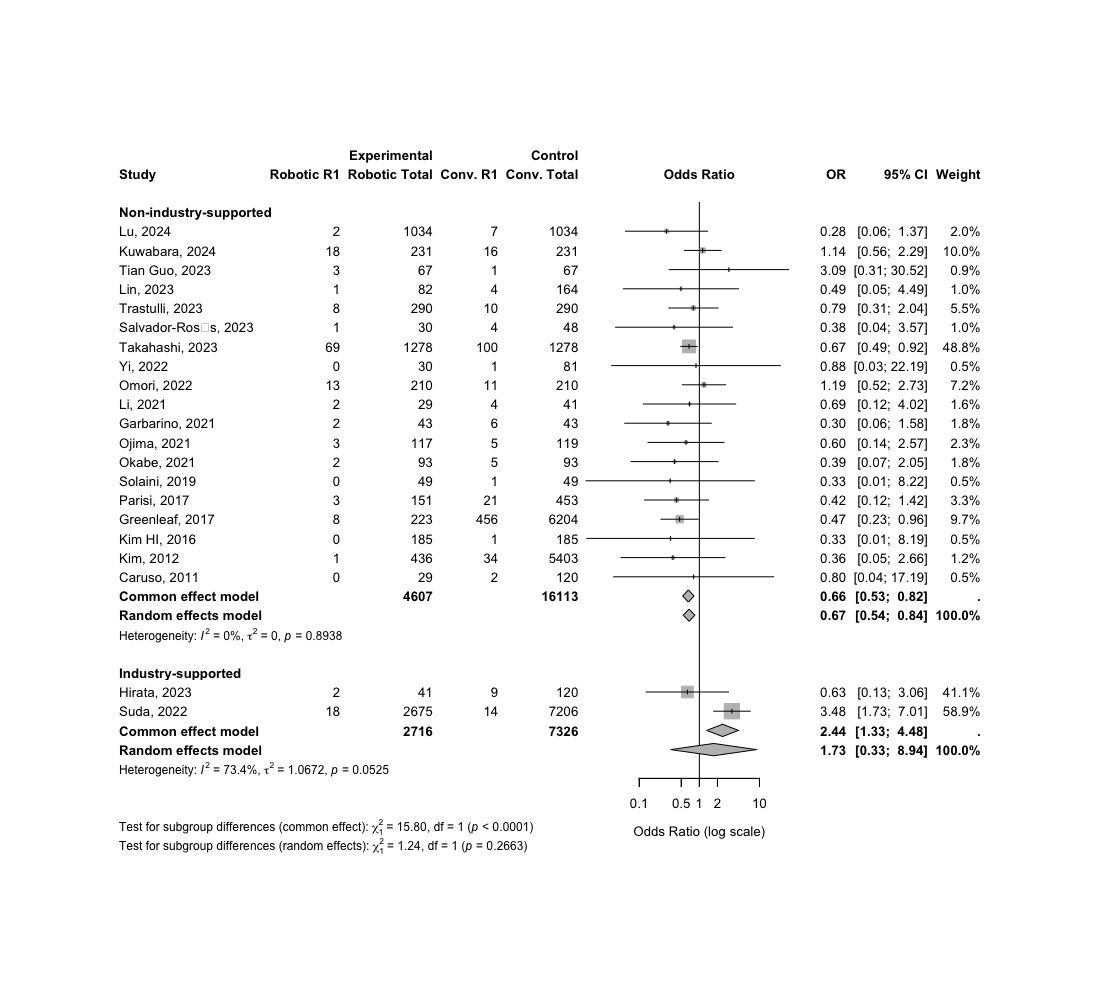


Figure S5 Bubble plot showing results of a meta-regression examining the association between the proportion of stage III patients in each study and the effect size (odds ratio) for R1 resection in robotic versus conventional gastrectomy.


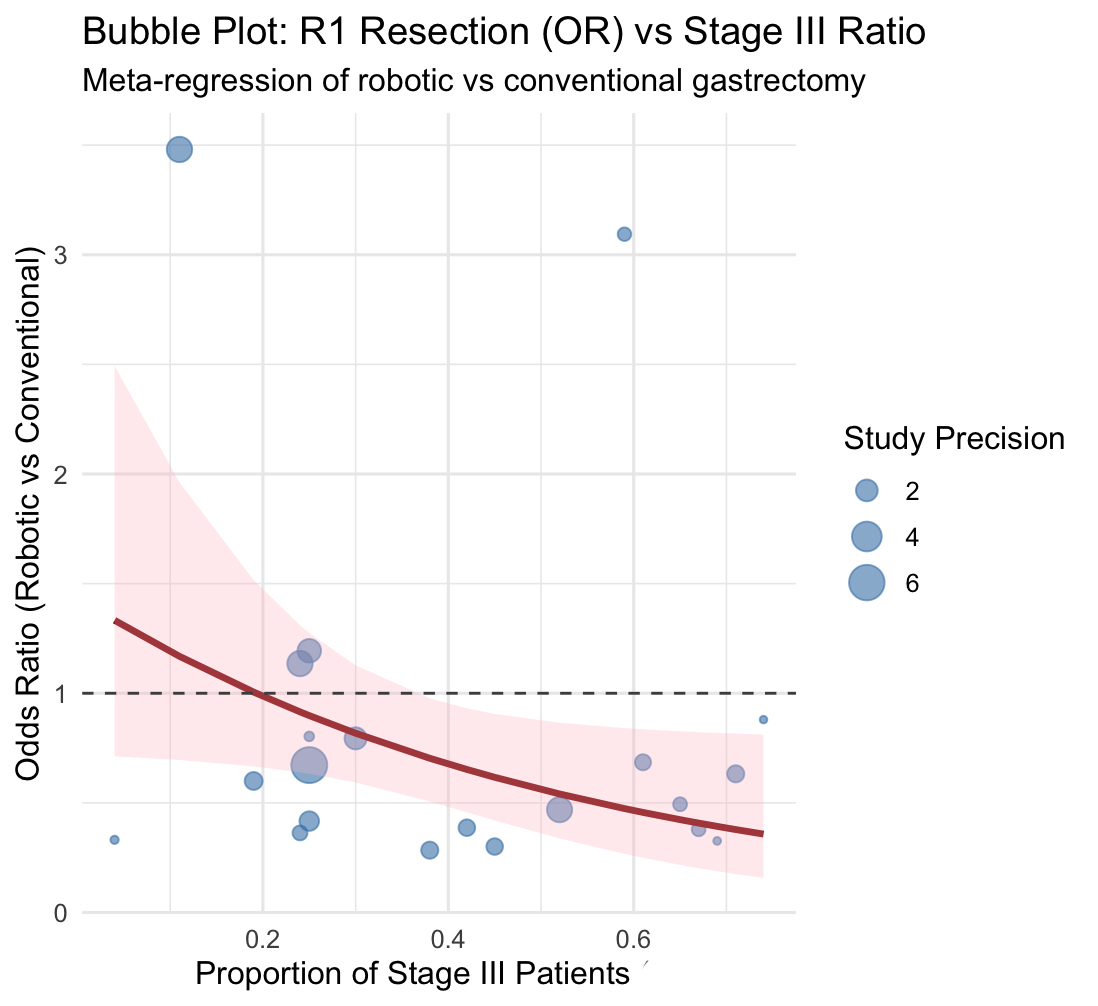


Figure S6 Forest plot of a subgroup analysis, stratified % of Stage III cancer, for R1 resection, showing odds ratios (ORs) with 95% confidence intervals (CIs) for each study comparing robotic (experimental arm) and conventional (control arm) gastrectomy.


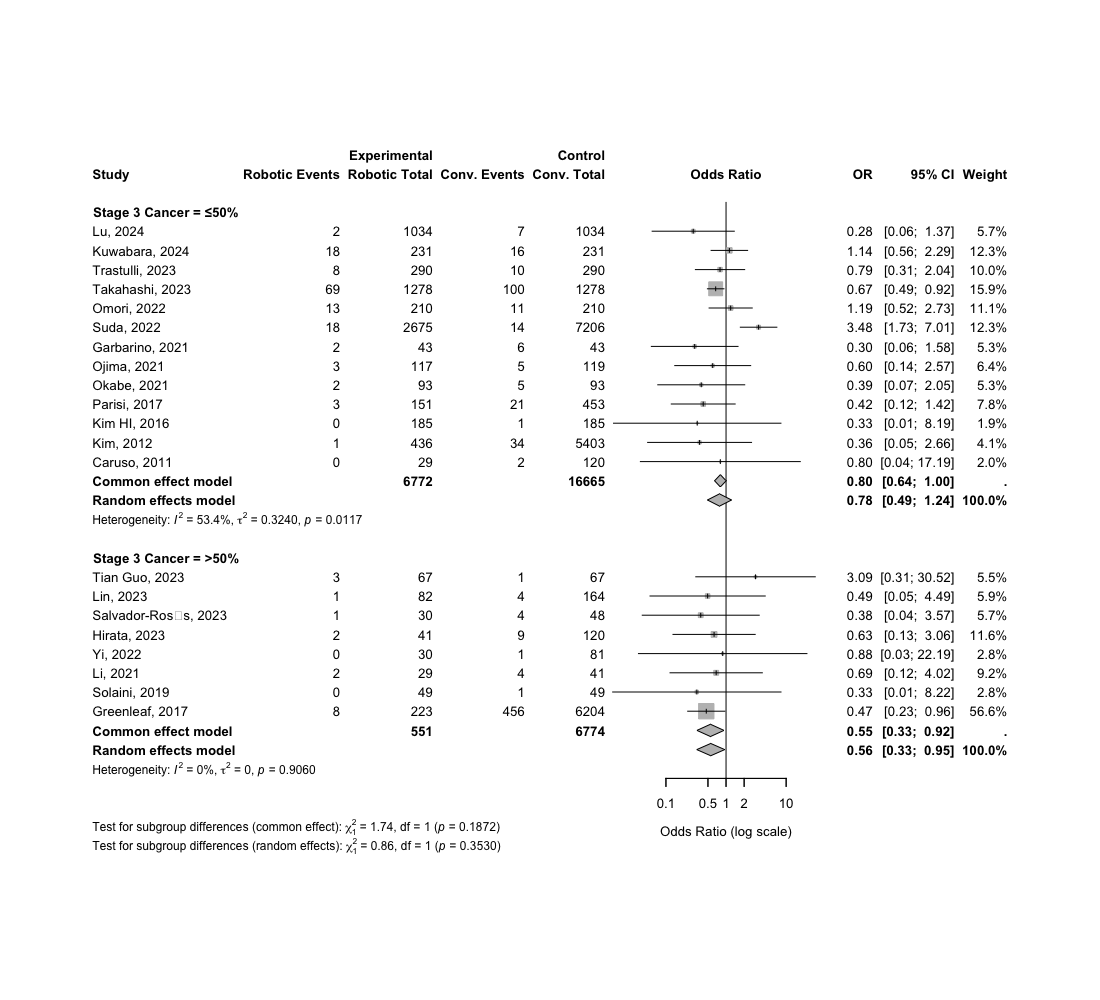


Figure S7 Forest plot of a subgroup analysis, stratified by geographical region showing odds ratios (ORs) with 95% confidence intervals (CIs) for each study comparing robotic (experimental arm) and conventional (control arm) gastrectomy. R1 Resections.

*
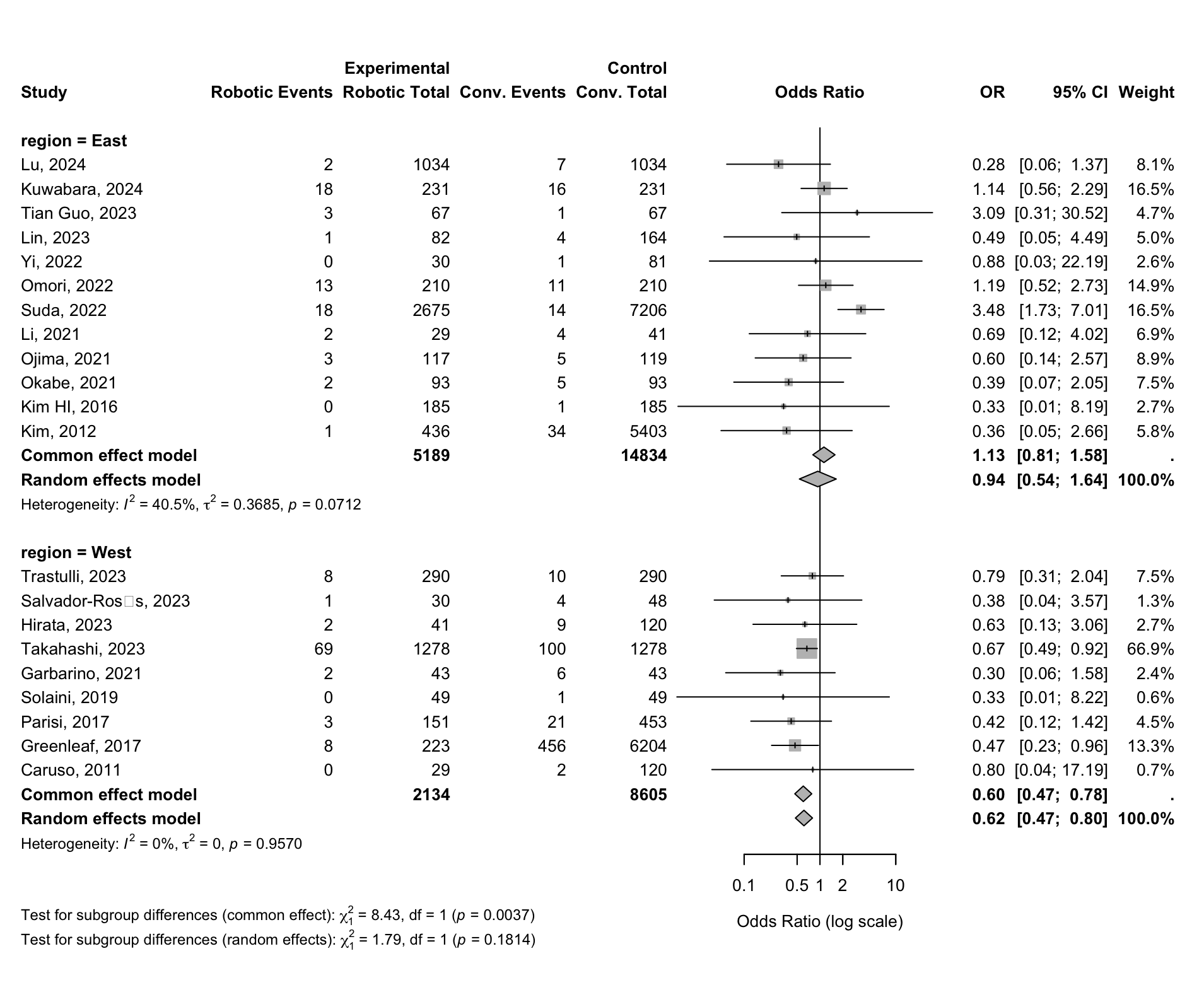
*

Figure S8-A Funnel plot of studies reporting positive resection margin (R1) comparing robotic vs conventional gastrectomy. No substantial asymmetry was observed.


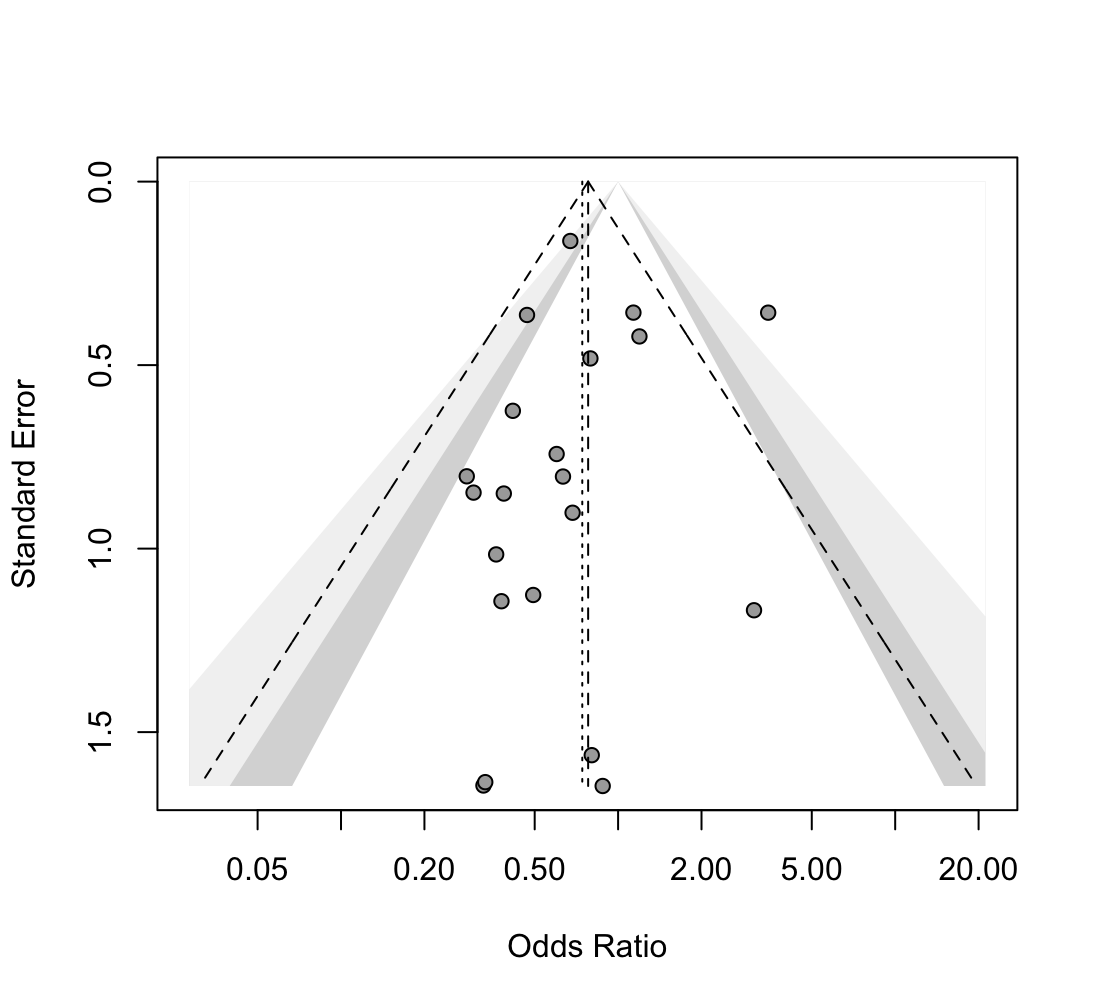


Figure S8-B Egger’s test for R1 funnel plot asymmetry was non-significant (t = - 0.73, df = 19, p = 0.4733), suggesting a low likelihood of small-study effects.
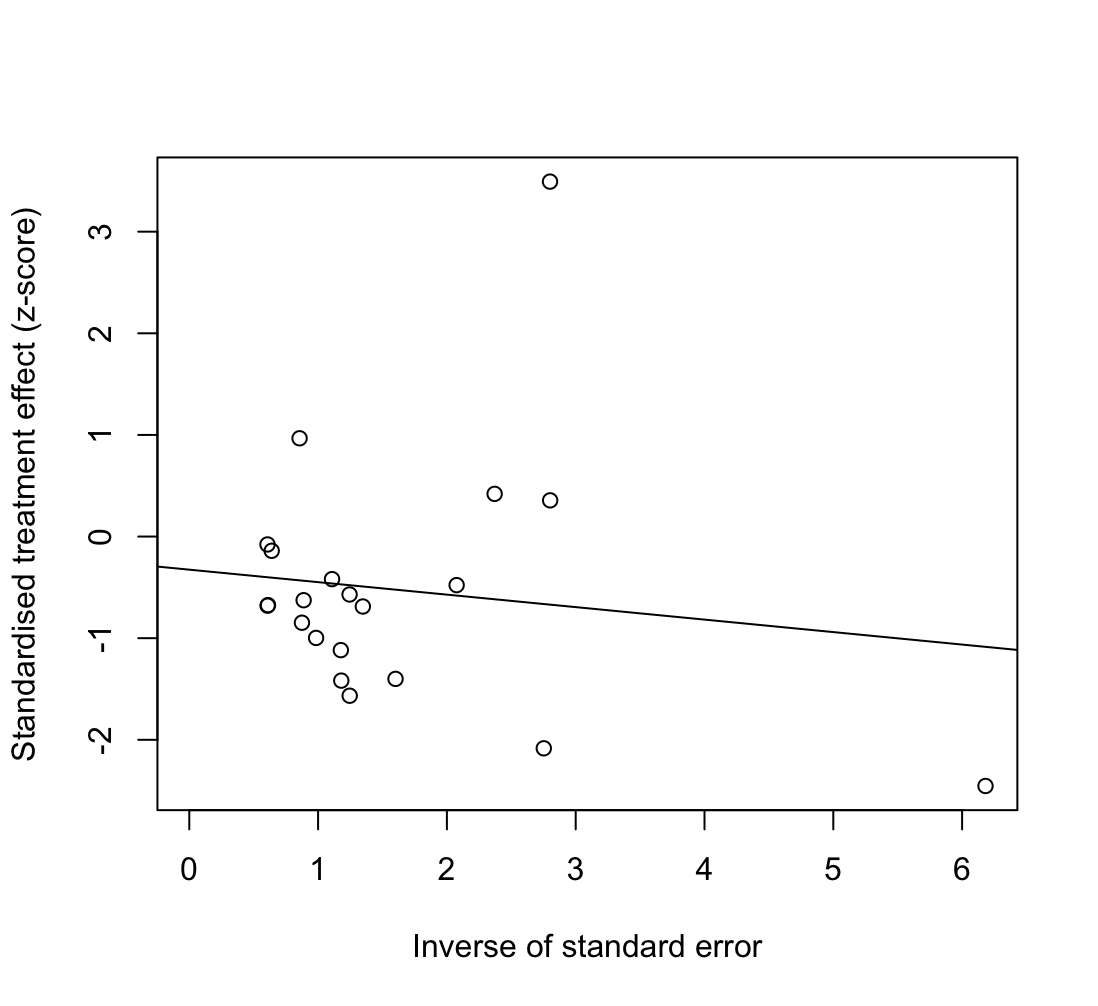


## Appendix S6: Secondary outcomes

### Clavien-Dindo grade III

Figure S9 Forest plot showing a random-effects meta-analysis of CD ≥ III complications comparing robotic and conventional gastrectomy, with odds ratios (ORs) and 95% confidence intervals (CI) for each study.
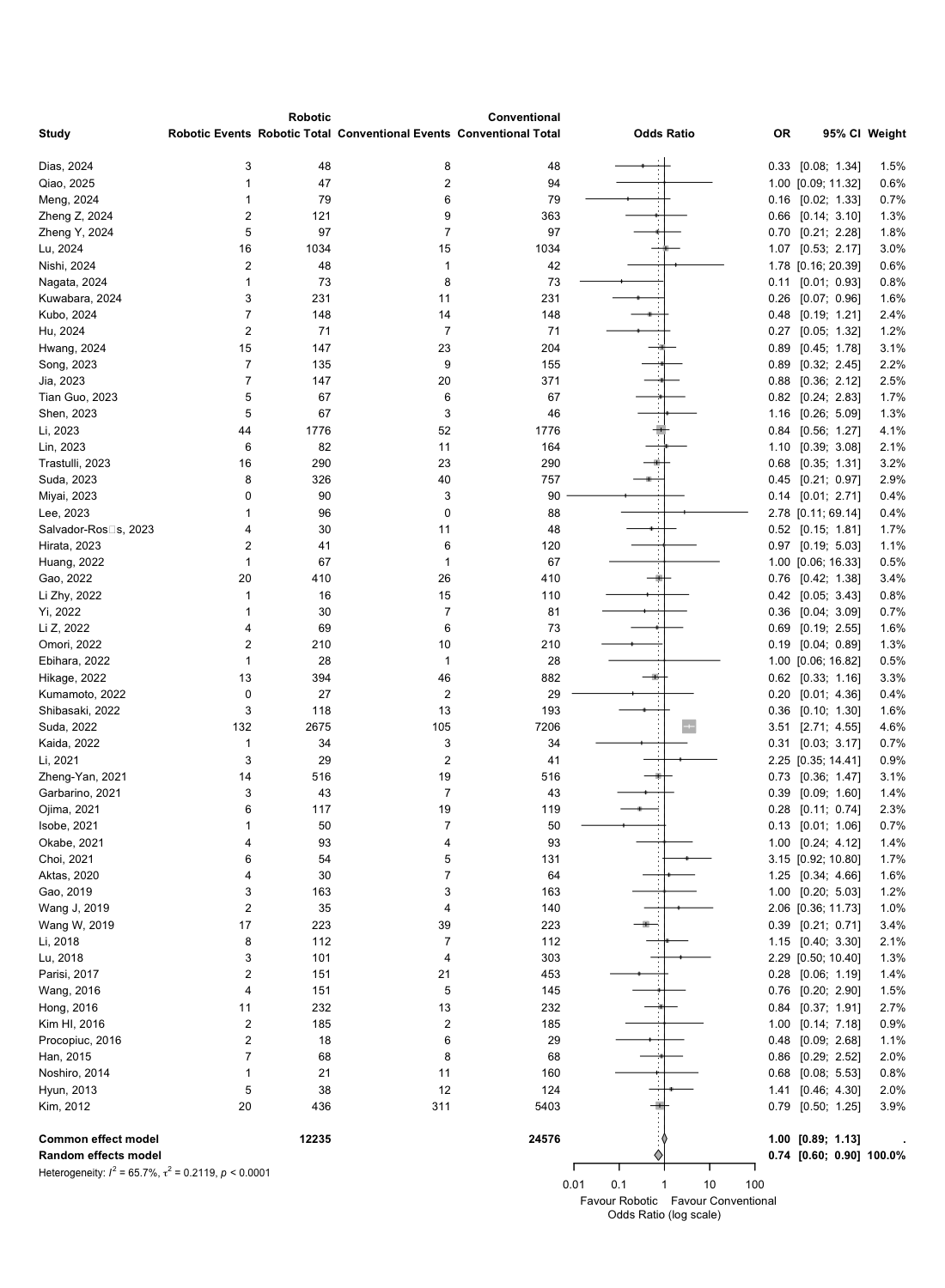


*Figure S10 Forest plot of a subgroup analysis, stratified by industry support, for Clavien-Dindo (CD) grade ≥ III complications, showing odds ratios (ORs) with 95% confidence intervals (CIs) for each study comparing robotic (experimental arm) and conventional (control arm) gastrectomy.*
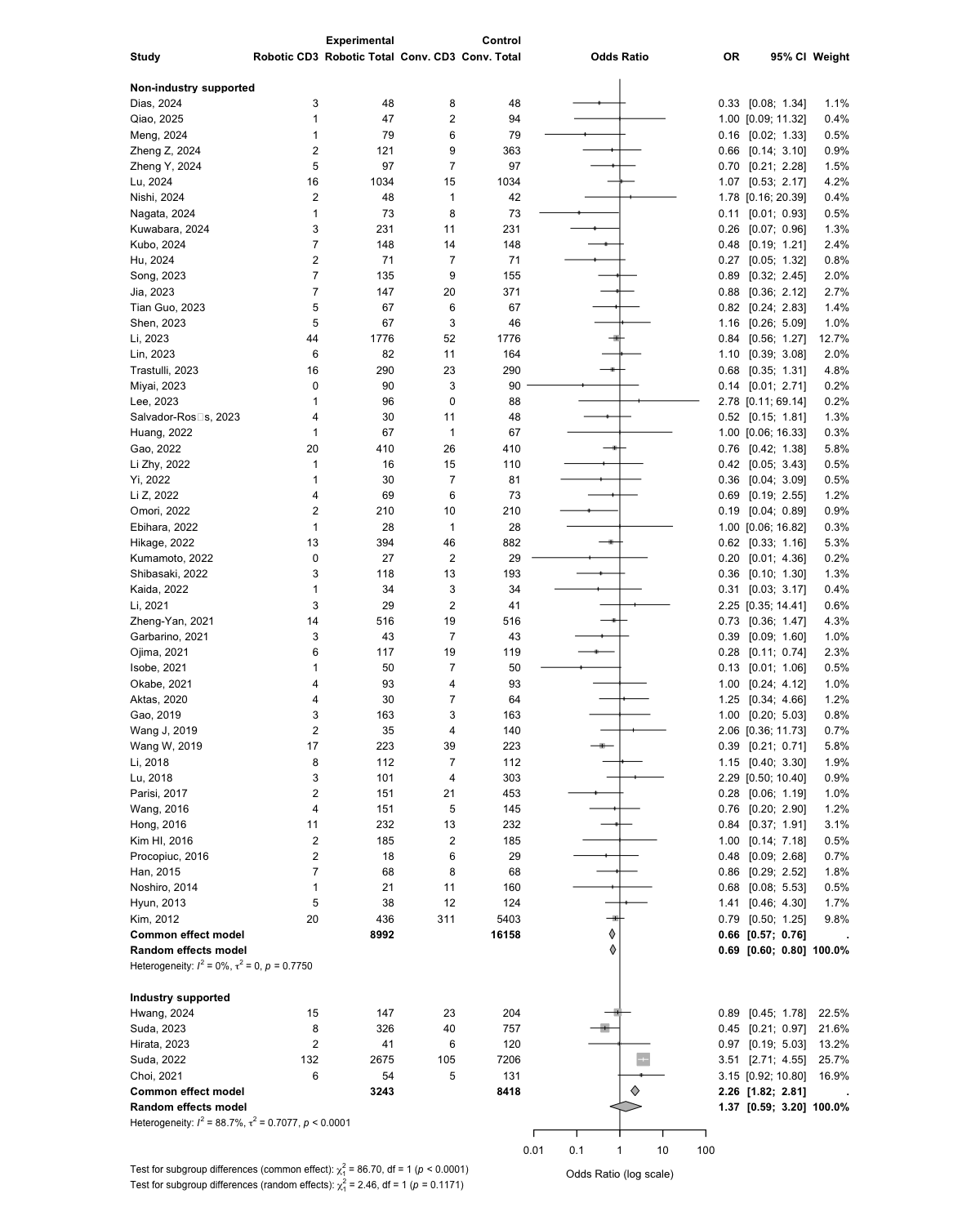


### Anastomotic leak

Figure S11 Forest plot showing a random-effects meta-analysis of anastomotic leak comparing robotic and laparoscopic gastrectomy, with odds ratios (ORs) and 95% confidence intervals (CI) for each study.


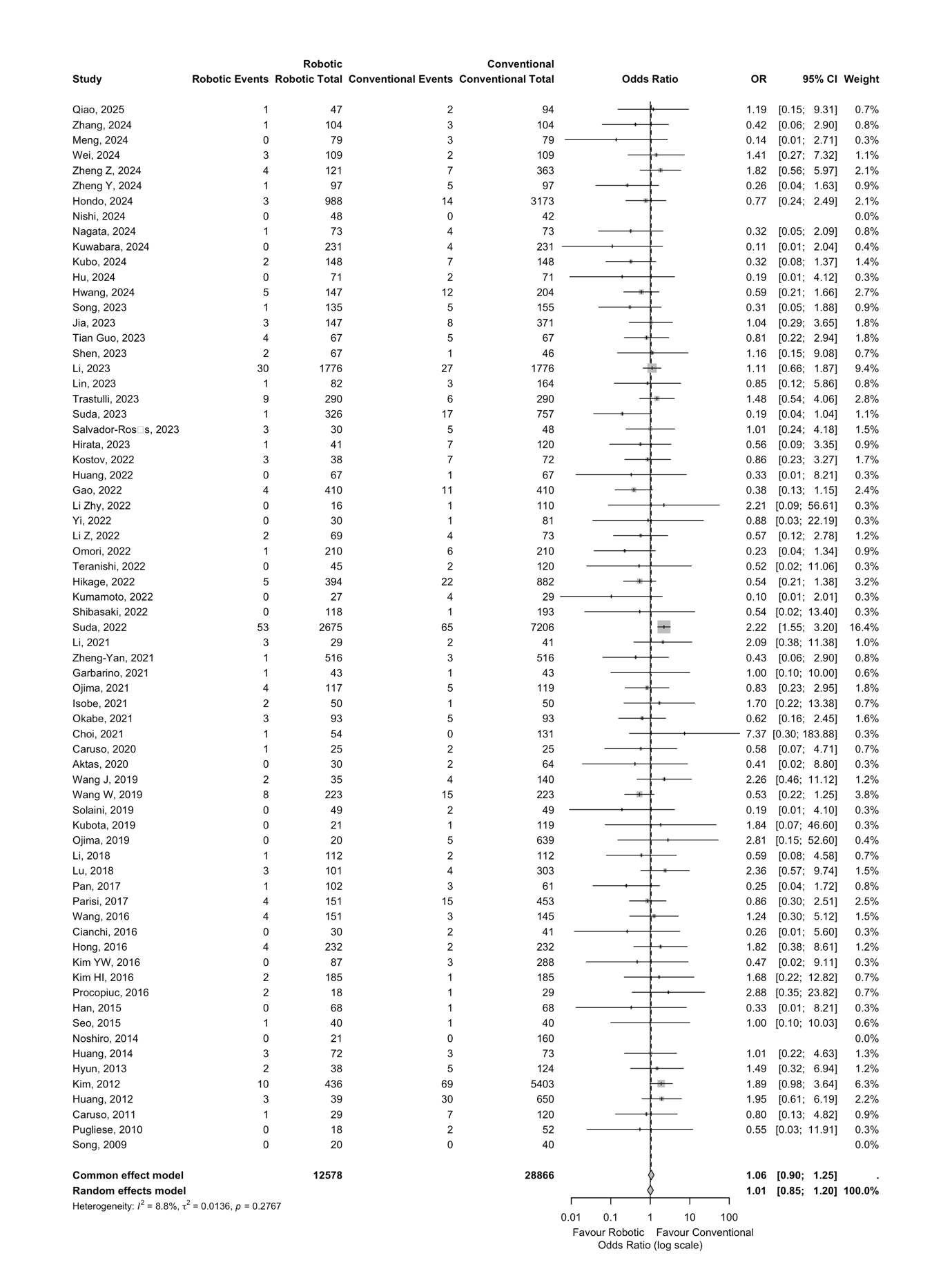


### Overall Complication

Figure S12 Forest plot showing a random-effects meta-analysis of overall complications comparing robotic and laparoscopic gastrectomy, with odds ratios (ORs) and 95% confidence intervals (CI) for each study.


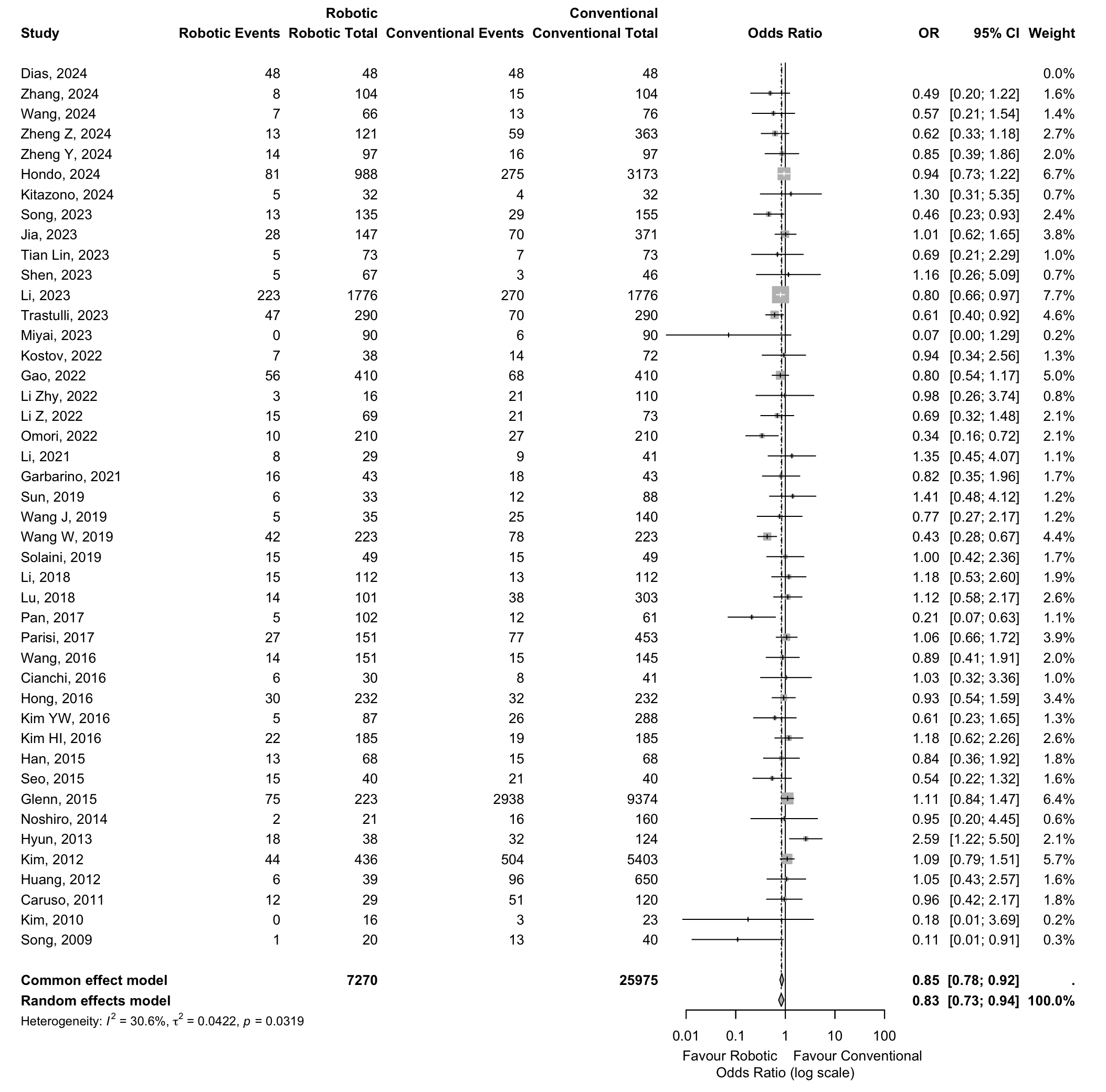


Table S4 GRADE summary table showing certainty of evidence for Clavien–Dindo grade ≥ III complications, and anastomotic leak.

### GRADE for secondary outcomes

| **Certainty assessment** | | | | | | | **№ of patients** | | **Effect** | | **Certainty** |
| --- | --- | --- | --- | --- | --- | --- | --- | --- | --- | --- | --- |
| **№ of studies** | **Study design** | **Risk of bias** | **Inconsistency** | **Indirectness** | **Imprecision** | **Other considerations** | **Robotic Gastrectomy** | **Conventional (Lap & Open) Gastrectomy** | **Relative (95% CI)** | **Absolute (95% CI)** |  |
| **Clavien-Dindo grade III or higher Complication** | | | | | | | | | | | |
| 58 | non-randomised studies | serious^a^ | serious^b^ | not serious | not serious | all plausible residual confounding would reduce the demonstrated effect^c^ | 463/12235 (3.8%) | 1037/24576 (4.2%) | **OR 0.74** (0.60 to 0.90) | **11 fewer per 1,000** (from 16 fewer to 4 fewer) | ⨁◯◯◯ Very low^a,b,c^ |
| **Anastomotic Leak** | | | | | | | | | | | |
| 66 | non-randomised studies | serious^a^ | not serious | not serious^d^ | serious^d^ | all plausible residual confounding would reduce the demonstrated effect | 211/12489 (1.7%) | 476/28624 (1.7%) | **OR 0.74** (0.58 to 0.93) | **4 fewer per 1,000** (from 7 fewer to 1 fewer) | ⨁◯◯◯ Very low^a,d^ |

| **Overall Complications** | | | | | | | | | | | | |
| --- | --- | --- | --- | --- | --- | --- | --- | --- | --- | --- | --- | --- |
| 44 | non-randomised studies | serious^a^ | serious^b^ | not serious | not serious | all plausible residual confounding would reduce the demonstrated effect | 1004/7270 (13.8%) | 5122/25975 (19.7%) | **OR 0.83** (0.73 to 0.94) | **28 fewer per 1,000** (from 45 fewer to 10 fewer) | ⨁◯◯◯ Very low^a,b^ |  |

| **CI:** confidence interval; **OR:** odds ratio  a. non-randomised studies with majority high or serious risk of bias  b. Statistical heterogeneity high  c. Asymmetrical funnel plot  d. Definition of clinical outcome vary between studies |
| --- |

## Appendix S7: ROBINS-I and ROB2 Risk of Bias

Figure S13 ROBINS-I risk of bias assessment for included non-randomised studies


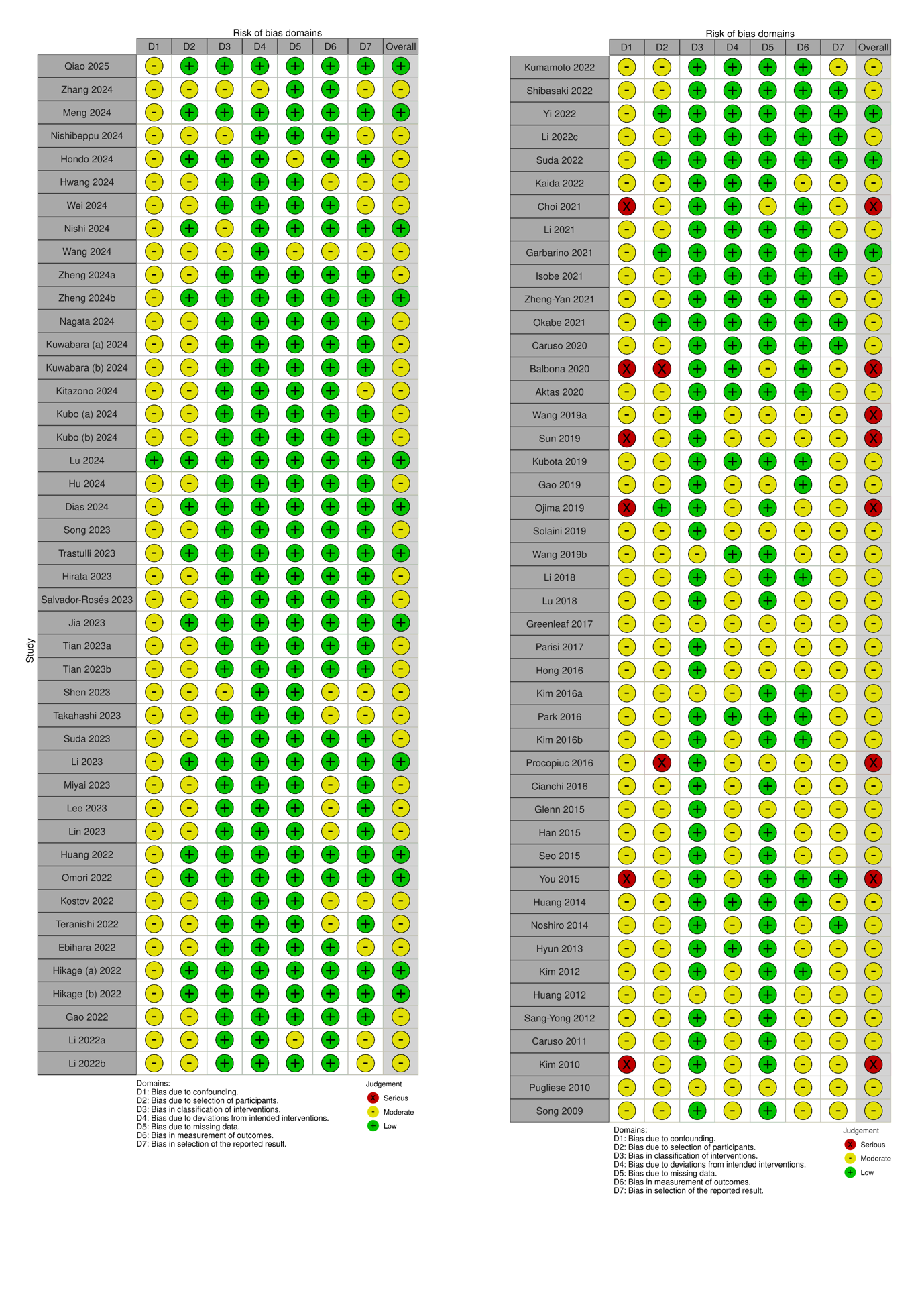


Figure S14 Summary of ROBINS-I risk of bias assessment for included non-randomised studies


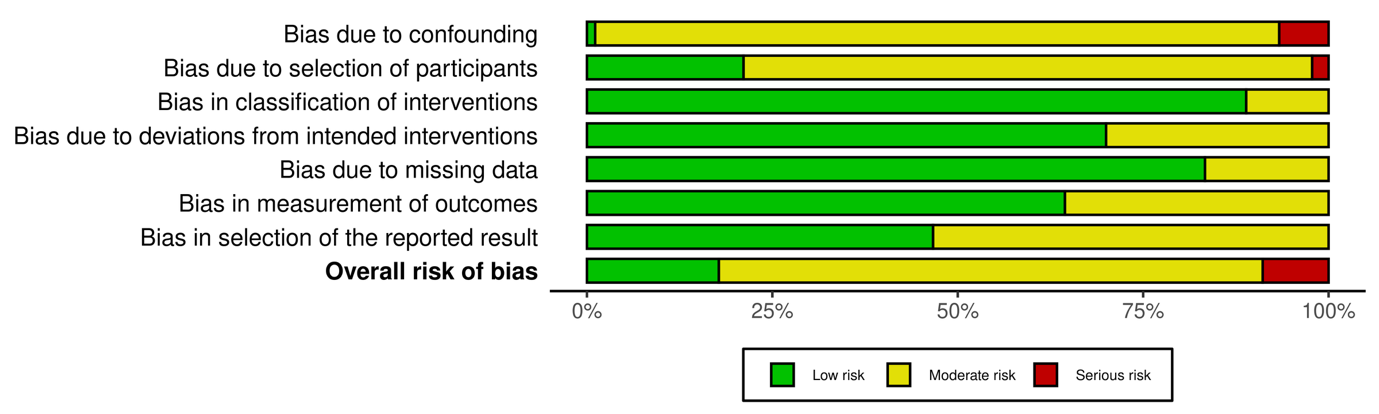


Figure S15 ROB2 risk of bias assessment for included randomised studies


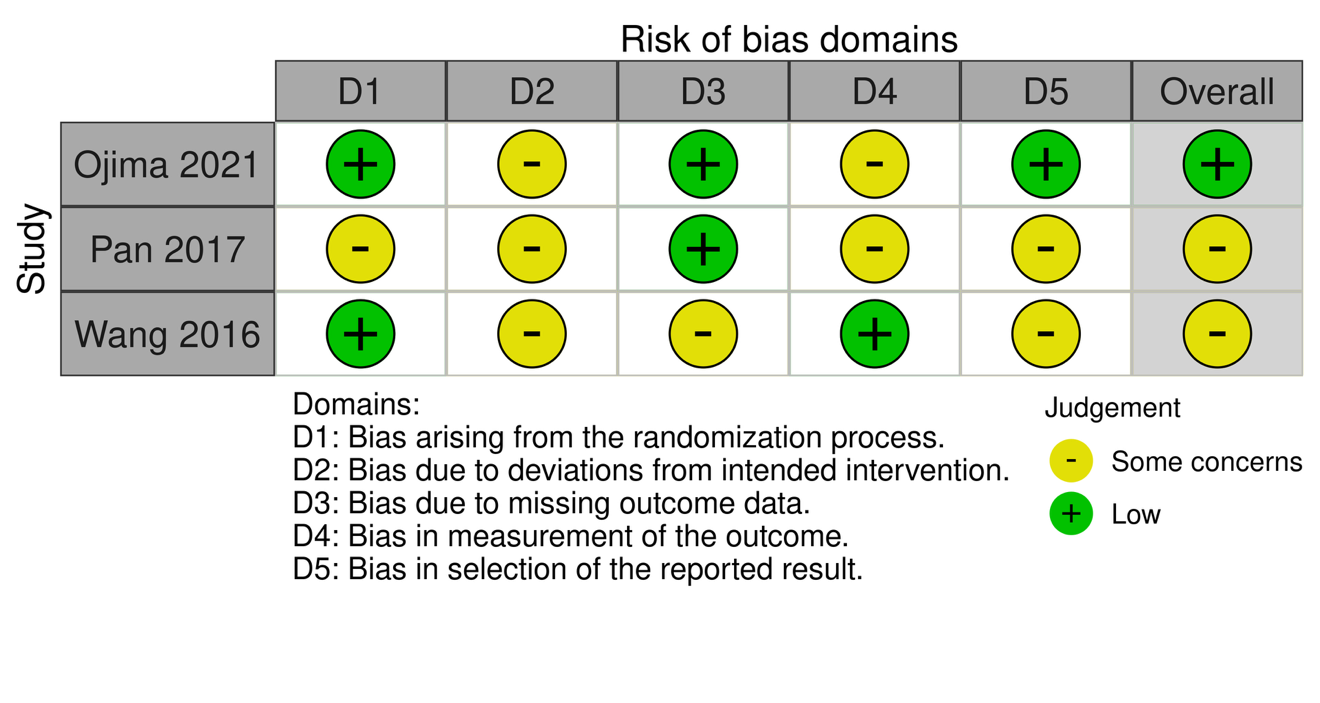


Figure S16 Summary of ROB2 risk of bias assessment for included randomised studies


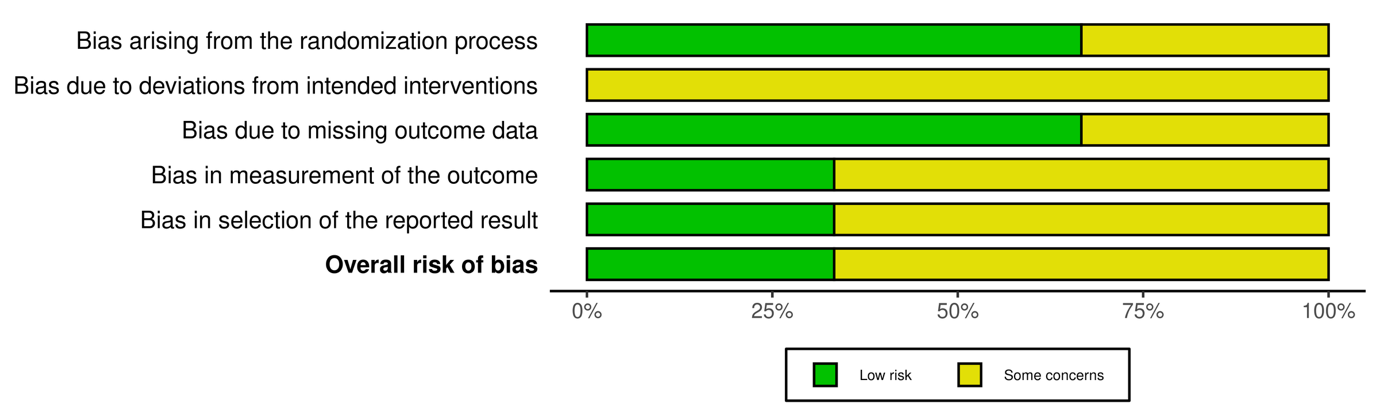

Supplement: zraf126_Supplementary_Data [file zraf126_supplementary_data.doc]
